# A novel in vitro model of trauma-induced endotheliopathy provides a platform for studying mechanisms of coagulopathy

**DOI:** 10.1016/j.bvth.2025.100087

**Published:** 2025-07-05

**Authors:** Jeries Abu-Hanna, Gang Xu, Gael B. Morrow, Lewis Timms, Naveed Akbar, Mike Laffan, Robin P. Choudhury, Nicola Curry

**Affiliations:** 1 Nuffield Division of Clinical Laboratory Sciences, Radcliffe Department of Medicine, University of Oxford, Oxford, United Kingdom; 2 Oxford Haemophilia and Thrombosis Centre, Oxford University Hospitals NHS Foundation Trust, Oxford, United Kingdom; 3 School of Pharmacy, Applied Sciences and Public Health, Robert Gordon University, Aberdeen, United Kingdom; 4 Division of Cardiovascular Medicine, Radcliffe Department of Medicine, University of Oxford, Oxford, United Kingdom; 5 Centre for Haematology, Department of Immunology and Inflammation, Imperial College London, London, United Kingdom

## Abstract

Trauma-induced coagulopathy (TIC) significantly contributes to trauma-related mortality, driven by dysregulated coagulation and fibrinolysis. Endotheliopathy of trauma (EoT) is central to TIC, yet its underlying mechanisms remain unclear. Current in vitro models fail to replicate the complex trauma environment, including hemorrhagic shock, tissue injury, and inflammation. This study aimed to develop a novel in vitro model of EoT that mimics key TIC features, enabling the investigation of endothelial contributions to TIC. Endothelial colony-forming cells (ECFCs) were exposed to trauma-relevant factors, including epinephrine, tumor necrosis factor α, interleukin 6, high mobility group box 1, hydrogen peroxide, and hypoxia. Endothelial injury markers (syndecan-1 and thrombomodulin), hemostatic protein expression, coagulation, and fibrinolysis were analyzed using enzyme-linked immunosorbent assay, immunofluorescence, global hemostasis assays, and RNA sequencing. Plasma from healthy donors and trauma patients was used to assess clinical relevance. Traumatized ECFCs exhibited progressive dysfunction, with early surface damage and sustained fibrinolytic dysregulation. Transcriptomic analysis showed activation of inflammatory pathways, metabolic shifts, and epigenetic changes. Surface expression of anticoagulant proteins decreased, whereas procoagulant tissue factor increased, heightening thrombogenic potential. Initially, traumatized ECFCs promoted fibrinolysis via thrombomodulin shedding but later secreted antifibrinolytic plasminogen activator inhibitor 1, mimicking the biphasic TIC phenotype. Plasma assays revealed thrombin generation and clot lysis changes similar to trauma patients. This in vitro model successfully replicates EoT and TIC-associated hemostatic imbalances, capturing the time-dependent evolution of endothelial dysfunction. It provides mechanistic insights into TIC and serves as a platform for testing targeted interventions to mitigate endothelial-driven coagulopathy in trauma.

## Key Points

• A novel in vitro model replicates trauma-induced endotheliopathy, capturing key coagulopathy features.
• The model exhibits temporal changes in hemostatic regulation, underpinned by transcriptomic, surface, and secretomic changes.

## Visual Abstract

**Figure:**
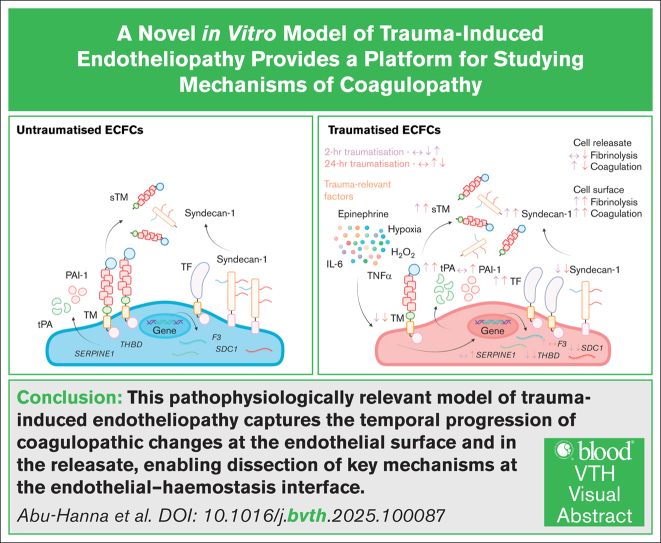

## Introduction

Traumatic injuries constitute a major global health concern, causing ∼4.4 million deaths annually.^1^ A key driver of trauma-related mortality is trauma-induced coagulopathy (TIC), an acquired dysfunction of hemostasis that occurs in 25% to 35% of severely injured patients.^2^^,^^3^ TIC encompasses a spectrum of coagulation disturbances, ranging from early-stage hypocoagulability and hyperfibrinolysis, leading to potentially fatal uncontrolled bleeding, to late-stage hypercoagulability and hypofibrinolysis, which contribute to widespread clot formation and multiple organ failure.^4^

Widespread endothelial injury and dysfunction, known as endotheliopathy of trauma (EoT), is thought to be a major contributor to TIC.^4^ EoT arises not only from direct injury but also from the body’s complex pathophysiological responses to the injury.^5^ Severe trauma causes bleeding and tissue damage, activating the sympathoadrenal system and inducing systemic inflammation, respectively.^6^ Blood loss leads to hypoperfusion and tissue hypoxia, whereas tissue damage releases damage-associated molecular patterns (DAMPs), such as high mobility group box 1 (HMGB1), which engage the innate immune system.^6^ Together, these processes drive endothelial dysfunction and may contribute to the progression of TIC.

The endothelium plays a crucial role in regulating hemostasis by expressing anticoagulant proteins, such as thrombomodulin (TM), syndecan-1, and tissue factor (TF) pathway inhibitor (TFPI), and releasing soluble fibrinolytic factors such as tissue plasminogen activator (tPA) and plasminogen activator inhibitor 1 (PAI-1).^7^ In healthy conditions, endothelial cells (ECs) protect blood vessels from thrombosis while promoting the formation of stable, localized fibrin clots that resist lysis at injury sites to prevent excessive bleeding.^5^ However, after traumatic injury, ECs undergo profound changes, leading to premature clot breakdown at injury sites and, paradoxically, the formation of clots within uninjured vessels.^5^ This is believed to contribute to multiple organ failure and death. The molecular mechanisms by which EoT contributes to TIC remain largely unknown.

In vitro studies using ECs have thus far failed to fully recapitulate the complex pathophysiological events (eg, epinephrine surge, poor tissue perfusion, tissue damage, and sterile inflammation) involved in EoT. Although the effects of hemorrhagic shock, caused by heavy blood loss and mimicked in vitro by hypoxia and epinephrine treatment, on EC function have been studied,^8^, ^9^, ^10^, ^11^ these conditions are not the sole drivers of EoT, and the synergistic effect of hemorrhagic shock, tissue injury, and sterile inflammation on the vascular endothelium remains unexplored. Moreover, in vitro studies have yet to assess the interaction of trauma-induced EC dysfunction with the coagulation and fibrinolysis systems.

In this study, we describe a novel in vitro model of EoT that mimics key features of TIC. First, we simulate key aspects of trauma pathophysiology to induce a state of endotheliopathy in endothelial colony-forming cells (ECFCs), surrogates for vascular ECs that can be noninvasively established from the peripheral blood of any consenting healthy donor or patient. Second, we explore the influence of the surface and releasate of experimentally “traumatized” ECFCs on fibrinolysis and coagulation and elucidate several molecular mechanisms through which EoT may contribute to TIC, illustrating the utility of the model in the detailed study of endothelial mechanisms of TIC. Last, we assess whether in vitro traumatization of ECFCs induces hemostatic changes in healthy plasma that mirror those observed in plasma from severely injured patients, to assess the clinical relevance and translational potential of the model.

## Methods

### ECFC generation and culture

ECFC cultures were established from 6 healthy donors (supplemental Table 1) as previously described, after donor consent and with Wales Research Ethics Committee approval (reference 20/WA/0313).^12^^,^^13^ We have assessed the endothelial identity and hemostatic behavior of ECFCs and confirmed their comparability to vessel-derived ECs (human umbilical vein ECs [HUVECs]; supplemental Figures 1-5**)**. For cell surface–based clot lysis (CL), thrombin generation (TG), and protein C activation assays, ECFCs were seeded in 96-well plates at 10 000 cells per well, cultured for 48 hours, and washed thrice with phosphate-buffered saline. For RNA extraction and supernatant collection, ECFCs were seeded in 6-well plates at 100 000 cells per well and grown to confluence.

### In vitro traumatization

“Traumatized” ECFCs were treated with a cocktail of trauma-related factors, at concentrations within ranges measured in trauma patients (supplemental Table 2) and guided by the data in supplemental Figure 6: 1 nM epinephrine^14^, ^15^, ^16^ (Sigma-Aldrich); 0.1 ng/mL tumor necrosis factor α^17^ (R&D Systems); 0.1 ng/mL interleukin 6^18^^,^^19^ (R&D Systems); 500 ng/mL HMGB1^20^^,^^21^ (Abcam); and 10-μM H_2_O_2_. The cells were then incubated for 2 and 24 hours in a hypoxia chamber with 1% O_2_. These conditions mimic the sympathoadrenal activation, inflammation, tissue damage–induced release of DAMPs and H_2_O_2_,^22^ and tissue hypoperfusion/hypoxia that occur in response to traumatic injuries. “Untraumatized” ECFCs were treated with the buffers used to reconstitute the recombinant proteins and incubated for 2 and 24 hours in a standard, humidified CO_2_ incubator with ∼20% O_2_. These traumatizing conditions did not significantly affect ECFC viability, as assessed by PrestoBlue cell viability assay (supplemental Figure 7).

### Enzyme-linked immunosorbent assay

Enzyme-linked immunosorbent assays for soluble TM (sTM; ab214029; Abcam), PAI-1 (ab108891; Abcam), syndecan-1 (DY2780; R&D Systems), and tPA (ab190812; Abcam) were performed following the manufacturer’s instructions.

### RNA isolation

ECFCs were lysed, and total RNA was extracted using the RNeasy Mini Kit (Qiagen) according to the manufacturer’s instructions.

### Bulk RNA-seq and analysis

Bulk RNA sequencing (RNA-seq) was performed by Novogene. Differential gene expression analysis was performed using DESeq2. Gene ontology (GO) enrichment analysis was conducted using the clusterProfiler R package. Volcano and dot plots were generated using the EnhancedVolcano and ggplot2 R packages.

### Immunocytochemistry

ECFCs were fixed with 4% paraformaldehyde for 15 minutes, permeabilized with 0.1% Triton X-100 for 5 minutes, blocked with 10% normal goat serum for 1 hour, and incubated overnight at 4°C with the primary antibodies listed in supplemental Table 3. After incubation with the primary antibodies, cells were incubated with the appropriate fluorescent secondary antibodies for 1 hour at room temperature and mounted with ProLong Gold Antifade Mountant containing the nuclear stain DAPI (4′,6-diamidino-2-phenylindole; Invitrogen). Imaging was performed using a Leica TCS SP8 confocal laser scanning microscope at 40× magnification.

### CL assay

CL assay was conducted as previously described^23^ in CRYOcheck pooled normal plasma (PNP; Precision BioLogic) and, in certain cases, plasma from individual healthy donors or patients with traumatic injuries from the Activation of Coagulation and Inflammation in Trauma study.^24^ Demographic and clinical data, including median age (40 years), median injury severity score (14), and time after injury (87 minutes), are summarized in supplemental Table 4. For releasate-based CL, 20% (volume-to-volume ratio) cell culture supernatants were added to PNP. Times to 50% CL (CLT_50_) in minutes were determined using the Shiny app for CL.^25^

### TG assay

TG was assessed using the calibrated automated thrombography method^26^ in PNP and, in some cases, plasma from individual healthy donors or trauma patients from the Activation of Coagulation and Inflammation in Trauma study.^24^ For cell releasate-based TG, 20% (volume-to-volume ratio) cell culture supernatants were added to PNP.

### Statistical analysis

All statistical analyses were performed on GraphPad Prism software (v10.4.1). Normality was assessed using the Shapiro-Wilk test. Most of the data were not normally distributed, and therefore, nonparametric tests were used. Data are expressed as median with interquartile range, with N denoting the number of biological replicates and n representing the number of technical replicates. Manny-Whitney and Wilcoxon tests were used to compare between unmatched and matched groups, respectively. *P* value <.05 was considered statistically significant.

### Study approval

This study was approved by the Wales Research Ethics Committee (reference 20/WA/0313).

## Results

### Recapitulating trauma conditions in vitro induces endotheliopathy in ECFCs

**Figure 1.**
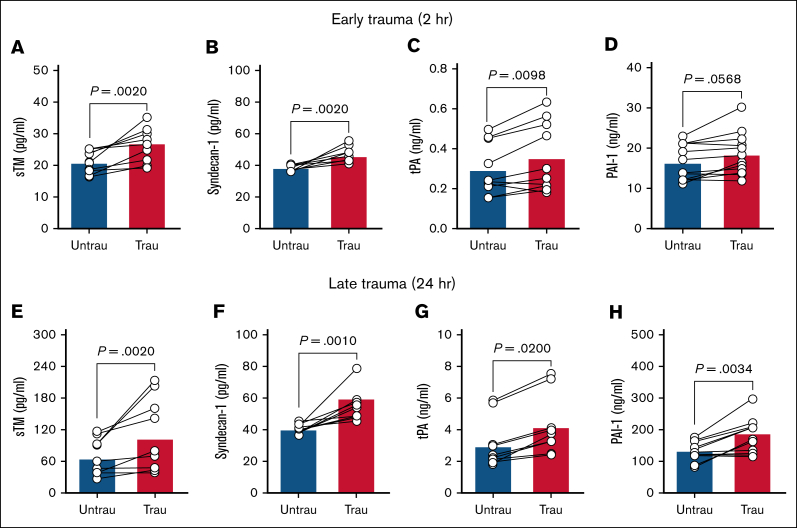
**Recapitulating trauma pathophysiology in vitro induces endotheliopathy in healthy ECFCs.** The levels of sTM (A), syndecan-1 (B), tPA (C), and PAI-1 (D) were quantified in the supernatants of ECFCs (biological replicate, N = 5; technical replicate, n = 2), cultured under untraumatizing (untrau) or traumatizing (trau) conditions for 2 hours. Similarly, the levels of sTM (E), syndecan-1 (F), tPA (G), and PAI-1 (H) were measured in the supernatants of ECFCs (biological replicate, N = 5; technical replicate, n = 2), cultured under untrau or trau conditions for 24 hours. Statistical analysis was performed using the Wilcoxon test for paired comparisons in panels A-H, with *P* value <.05 considered statistically significant.

In trauma patients, elevated circulating levels of sTM and syndecan-1 indicate extensive endothelial injury and damage.^27^^,^^28^ Additionally, early elevations in plasma tPA contribute to a hyperfibrinolytic state, whereas PAI-1 levels increase over time after injury, promoting late-stage hypofibrinolysis.^29^^,^^30^ To confirm whether our in vitro traumatization protocol induces endothelial changes such as those observed in trauma patients, we assessed endothelial dysfunction by measuring key endothelial damage and fibrinolytic markers in ECFC supernatants after trauma exposure. After 2 hours of traumatization, there was evidence of early endothelial damage, as indicated by increased levels of sTM and syndecan-1 (Figure 1A-B). This was accompanied by elevated tPA (Figure 1C), although PAI-1 levels remained unchanged (Figure 1D). By 24 hours, endothelial damage persisted, with further increases in sTM and syndecan-1 (Figure 1E-F). At this later stage, both tPA and PAI-1 were significantly elevated (Figure 1G-H), indicating a shift in fibrinolytic balance. These findings suggest that ECFC traumatization induces progressive endothelial dysfunction, beginning with early endothelial surface damage and evolving into broader dysregulation of fibrinolytic markers over time.

### In vitro traumatization induces a global trauma-like phenotype in ECFCs

**Figure 2.**
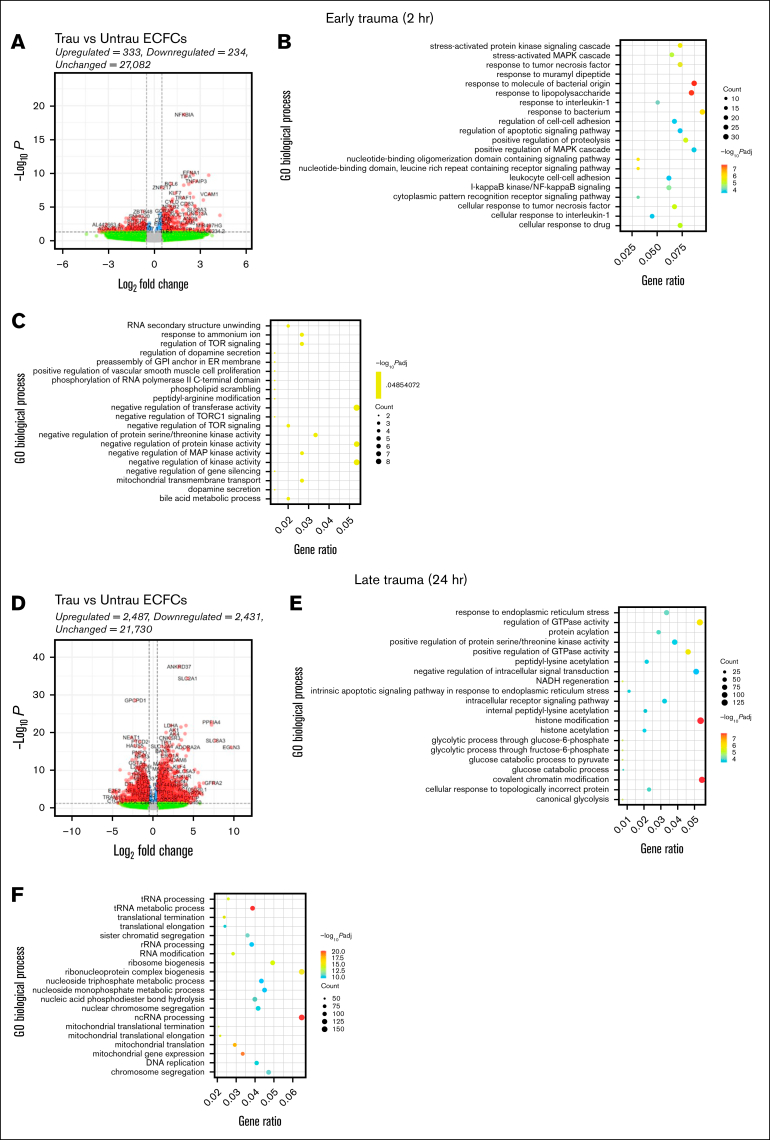
**In vitro traumatization induces transcriptional changes associated with endothelial dysfunction in healthy ECFCs.** Bulk RNA-seq was performed on healthy ECFCs cultured under untrau or trau conditions for 2 and 24 hours, representing the early and late effects of trauma, respectively. (A) Volcano plot showing differentially expressed genes (DEGs) in 2-hour trau ECFCs compared to 2-hour untrau ECFCs, with –log_10_*P* and log_2_ fold change thresholds set to 1.3 (*P* = .05) and 1 (fold change = 2), respectively. Dot plots showing the top 20 significantly upregulated (B) and downregulated (C) GO biological processes in 2-hour trau ECFCs. (D) Volcano plot showing DEGs in 24-hour trau ECFCs compared to 24-hour untrau ECFCs with –log_10_*P* and log_2_ fold change thresholds set to 1.3 and 1, respectively. Dot plots showing the top 20 significantly upregulated (E) and downregulated (F) GO biological processes in 24-hour trau ECFCs. GPI, glycosylphosphatidylinositol; NADH, reduced nicotinamide adenine dinucleotide; ncRNA, noncoding RNA; rRNA, ribosomal RNA; tRNA, transfer RNA.

Trauma patients experience a rapid and robust inflammatory response, which is essential for initiating tissue repair and deploying immune defenses.^31^ After this initial inflammatory response, trauma patients exhibit a pronounced metabolic shift toward a catabolic and glycolytic phenotype to meet the immediate energy demands of immune and reparative processes.^31^^,^^32^ To examine whether our in vitro trauma conditions induce similar responses and phenotypic changes in healthy ECFCs, we performed bulk RNA-seq followed by differential gene expression and GO enrichment analyses. By 2 hours, transcriptional changes were modest, with 333 genes upregulated and 234 downregulated. GO analysis of the upregulated genes highlighted biological processes involved in stress (eg, stress-activated protein kinase signaling and MAPK signaling**)** and inflammatory responses (eg, I-κB kinase/NF-κB signaling, response to tumor necrosis factor, interleukin 1, and bacterial stimuli, and leukocyte cell-cell adhesion; Figure 2B). Conversely, the downregulated genes were associated with processes such as RNA secondary structure unwinding, regulation of target of rapamycin (TOR) signaling, and mitochondrial transport (Figure 2C). These processes suggest a reduction in metabolic and transcriptional regulatory activity, potentially indicative of early cellular stress or dysfunction.

By 24 hours, transcriptional changes intensified, with 2487 genes upregulated and 2431 downregulated (Figure 2D). GO analysis of these genes revealed significant enrichment in pathways related to endoplasmic reticulum stress, protein regulation, glycolysis, and intrinsic apoptotic signaling (Figure 2E), suggesting that prolonged exposure to trauma-like conditions may contribute to endothelial dysfunction through stress-related apoptotic mechanisms. Interestingly, upregulation of histone modification and chromatin remodeling pathways indicates potential epigenetic reprogramming, aligning with findings that trauma-induced stress can drive epigenetic changes in ECs. Downregulated genes at this stage were associated with mitochondrial function and ribosome biogenesis, with significant suppression of pathways such as transfer RNA metabolism and mitochondrial translation (Figure 2F). This suppression suggests impaired metabolic activity and protein synthesis, which have been previously reported to occur in trauma patients.

Collectively, these results demonstrate that our in vitro traumatization protocol induces a phenotype in ECFCs similar to that observed in major trauma patients, underscoring the disease relevance of our model. This phenotype is characterized by early inflammatory activation, subsequent glycolytic and catabolic reprogramming, and potential epigenetic reprogramming.

### In vitro traumatization alters the hemostatic transcriptome of ECFCs

**Figure 3.**
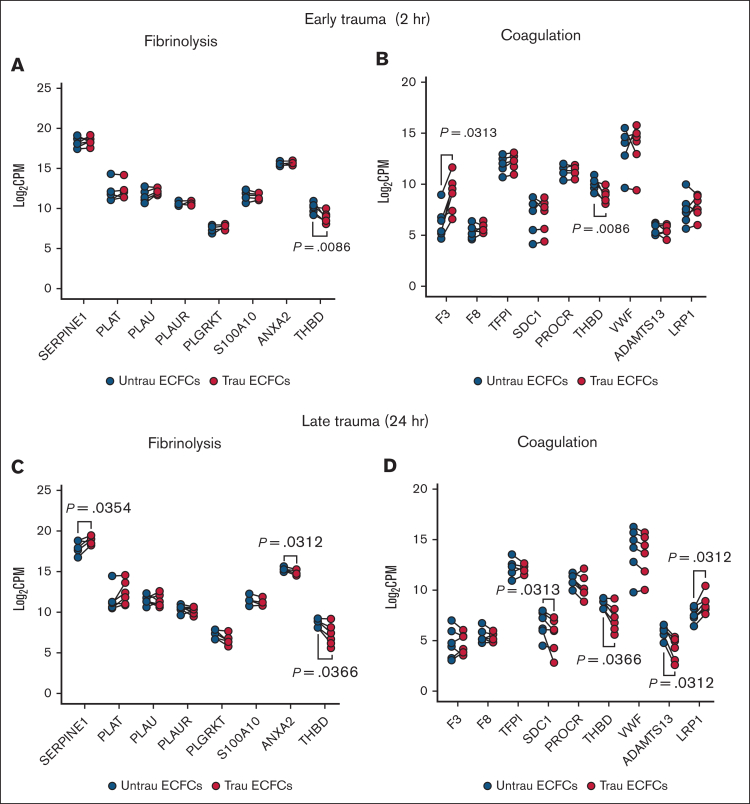
**In vitro traumatization induces changes in the hemostatic transcriptome of healthy ECFCs.** Bulk RNA-seq analysis revealed differential expression of key endothelial hemostatic genes between ECFCs, cultured under untrau and trau conditions for 2 hours or 24 hours. (A-B) Log_2_ transformed CPM values for transcripts of fibrinolysis-associated (A) and coagulation-associated (B) genes in 2-hour untrau or trau ECFCs. (C-D) Log_2_ transformed CPM values for transcripts of fibrinolysis-associated (C) and coagulation-associated (D) genes in 24-hour untrau or trau ECFCs. Statistical analysis was performed using the Wilcoxon test for paired comparisons in panels A-D, with *P* value <.05 considered statistically significant. CPM, counts per million.

Delving into the hemostatic transcriptome, bulk RNA-seq analysis revealed significant changes in the expression of key endothelial hemostatic genes after in vitro traumatization of ECFCs for 2 and 24 hours. After 2 hours of traumatization, fibrinolysis-associated genes showed minimal alterations, with only *THBD* (encoding TM) being downregulated (Figure 3A). In contrast, coagulation-associated genes (Figure 3B) exhibited broader changes, including increased expression of the procoagulant gene *F3* (encoding TF) and reduced expression of the anticoagulant gene *THBD*, suggesting an early procoagulant shift. By 24 hours of traumatization, fibrinolysis-related transcripts (Figure 3C) displayed more pronounced alterations, with the antifibrinolytic genes *SERPINE1* (encoding PAI-1) and *THBD* being upregulated and downregulated, respectively, and the profibrinolytic gene *ANXA2* (encoding annexin A2, a tPA and plasminogen receptor that acts in concert with S100A10^33^, ^34^, ^35^) being downregulated, indicating a time-dependent modulation of the endothelial fibrinolytic response. Meanwhile, coagulation-associated genes (Figure 3D) continued shifting toward a procoagulant profile, with the anticoagulant genes *SDC1* (encoding syndecan-1), *THBD*, and *ADAMTS13* (encoding the von Willebrand factor–cleaving metalloprotease ADAMTS13^36^) being downregulated. Interestingly, the F8-scavenging gene *LRP1*^37^ was upregulated after 24 hours of traumatization. These findings suggest that endothelial traumatization may contribute to TIC not only through the damage of surface hemostatic proteins but also via transcriptional alterations, highlighting a novel mechanism of hemostatic dysregulation.

### In vitro traumatization remodels the hemostatically active surface of ECFCs

**Figure 4.**
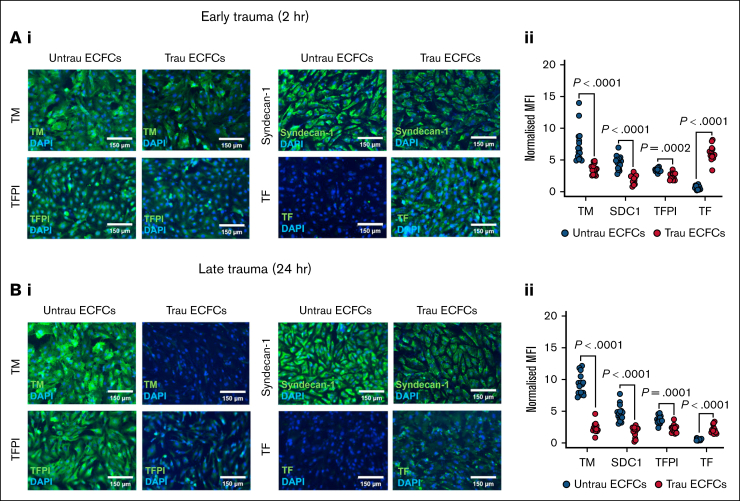
**In vitro traumatization remodels the hemostatically active surface of ECFCs.** (A) Immunofluorescent staining of ECFCs (biological replicate, N = 5; technical replicate, n = 3), cultured under untrau or trau conditions for 2 hours, for surface TM, syndecan-1, TFPI, and TF (i), with the corresponding MFI values normalized to the MFI of the nuclear stain DAPI (ii). (B) Immunofluorescent staining of ECFCs (biological replicate, N = 5; technical replicate, n = 3), cultured under untrau or trau conditions for 24 hours, for surface TM, syndecan-1, TFPI, and TF (i), with the corresponding MFI values normalized to the MFI of the nuclear stain DAPI (ii). Data are presented as median with interquartile range. Statistical analysis was performed using the Wilcoxon test for paired comparison in panels Aii,Bii; with *P* value <.05 considered statistically significant. MFI, mean fluorescence intensity.

To assess the impact of in vitro traumatization on the hemostatic proteins decorating the surface of ECFCs, immunofluorescent staining was performed for TM, syndecan-1, TFPI, and TF after 2 and 24 hours. After 2 hours of traumatization (Figure 4A), surface expression of TM, syndecan-1, and TFPI was reduced compared to untraumatized ECFCs, whereas TF expression was increased (Figure 4Ai-ii), indicating an early procoagulant shift. By 24 hours of traumatization (Figure 4Bi-ii), these alterations became more pronounced. The surface expression of TM, syndecan-1, and TFPI remained significantly reduced, whereas TF expression was further elevated compared to untraumatized ECFCs. Together, these results indicate that in vitro traumatization alters the composition of the hemostatic surface of ECFCs by downregulating anticoagulant proteins (TM, syndecan-1, and TFPI) and upregulating the procoagulant TF, thereby enhancing their thrombogenic potential.

### In vitro traumatization alters the fibrinolytic potential of the surface and releasate of ECFCs

**Figure 5.**
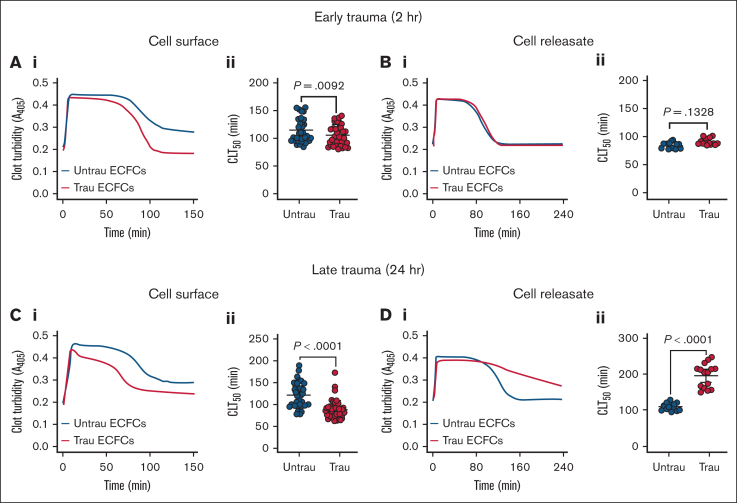
**In vitro traumatization accelerates tPA-induced CL on the surface of healthy ECFCs while markedly delaying it in their releasates.** (A) Clot formation and lysis in PNP over time in the presence of healthy ECFCs (biological replicate, N = 5; technical replicate, n = 6), cultured under untrau or trau conditions for 2 hours (i), with associated CLT_50_ (ii). (B) Clot formation and lysis in PNP incorporating cell culture supernatants from 2-hour untrau or trau ECFCs (biological replicate, N = 5; technical replicate, n = 3) over time (i), with derived CLT_50_ (ii). (C) Clot formation and lysis in PNP over time in the presence of healthy ECFCs (biological replicate, N = 5; technical replicate, n = 6), cultured under untrau or trau conditions for 24 hours (i), with associated CLT_50_ (ii). (D) Clot formation and lysis in PNP incorporating cell culture supernatants from 24-hour untrau or trau ECFCs (biological replicate, N = 5; technical replicate, n = 3) over time (i), with derived CLT_50_ (ii). Data are presented as median with interquartile range. Statistical analysis was performed using the Wilcoxon test for paired comparisons in panels Aii,Bii,Cii,Dii, with *P* value <.05 considered statistically significant.

Given the in vitro traumatization-induced shedding and downregulation of surface antifibrinolytic proteins (eg, TM), along with the time-dependent increased secretion of both profibrinolytic (eg, tPA) and antifibrinolytic proteins (eg, PAI-1), we hypothesized that ECFCs would develop a profibrinolytic surface while simultaneously generating an antifibrinolytic releasate. After 2 hours of traumatization, ECFCs exhibited a modest profibrinolytic effect, as evidenced by a significant reduction in CL time (CLT_50_) on their surface (Figure 5A). However, ECFC-derived releasates at this time point had no significant effect on fibrinolysis (Figure 5B). With prolonged traumatization (24 hours), ECFCs displayed a more pronounced profibrinolytic shift, further shortening CLT_50_ (Figure 5C). In contrast, releasates collected at this stage induced a strong antifibrinolytic response, significantly prolonging CLT_50_ (Figure 5D). These results suggest that ECFC traumatization leads to dynamic fibrinolytic changes, with early profibrinolytic activity at the cell surface due to rapid TM shedding and downregulation, followed by a later antifibrinolytic effect in the surrounding environment, likely driven by increased PAI-1 secretion over time.

### In vitro traumatization alters the thrombogenicity of the surface and releasate of ECFCs

**Figure 6.**
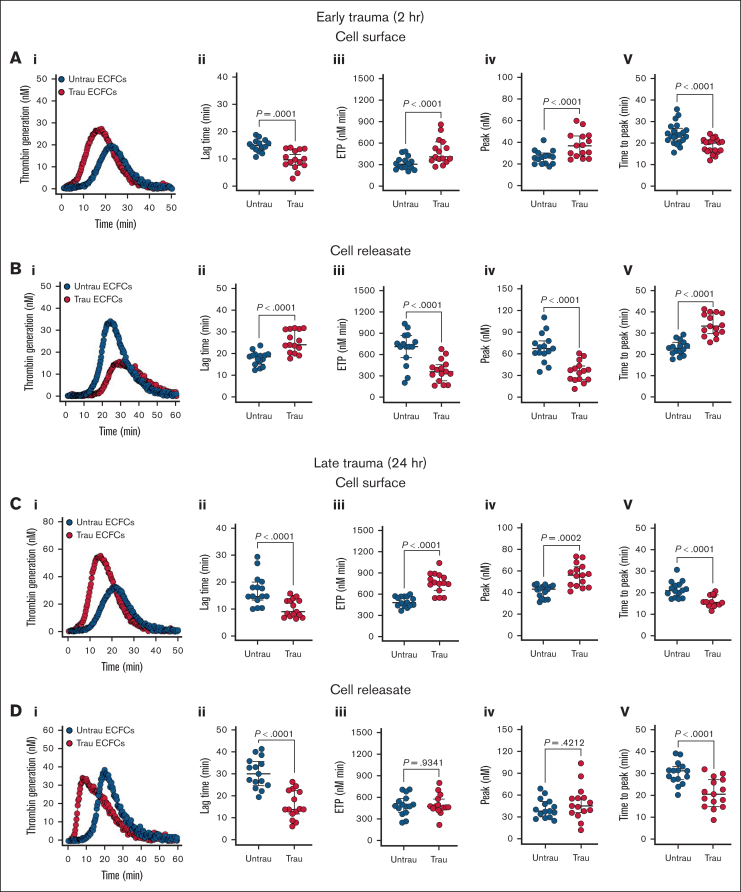
**In vitro traumatization promotes TG on the surface of healthy ECFCs.** (A) TG in PNP in the presence of healthy ECFCs (biological replicate, N = 5; technical replicate, n = 3), cultured under untrau or trau conditions for 2 hours (i), with TG parameters: lag time (ii), ETP (iii), peak thrombin (iv), and time to peak thrombin (v). (B) TG in PNP incorporating cell culture supernatants from 2-hour untrau or trau ECFCs (biological replicate, N = 5; technical replicate, n = 3) over time (i), with derived parameters: lag time (ii), ETP (iii), peak thrombin (iv), and time to peak thrombin (v). (C) TG in PNP in the presence of healthy ECFCs (biological replicate, N = 5; technical replicate, n = 3), cultured under untrau or trau conditions for 24 hours (i), with derived parameters: lag time (ii), ETP (iii), peak thrombin (iv), and time to peak thrombin (v). (D) TG in PNP incorporating cell culture supernatants from 24-hour untrau or trau ECFCs (biological replicate, N = 5; technical replicate, n = 3) over time (i), with derived parameters: lag time (ii), ETP (iii), peak thrombin (iv), and time to peak thrombin (v). Data are presented as median with interquartile range. Statistical analysis was performed using the Wilcoxon test for paired comparisons in panels Aii-v,Bii-v,Cii-v,Dii-v; *P* value <.05 was considered statistically significant.

Given the in vitro traumatization-induced shedding and/or downregulation of surface anticoagulant proteins (eg, TM, syndecan-1, and TFPI), along with the upregulation of surface procoagulant proteins (eg, TF), we hypothesized that ECFCs would develop a prothrombogenic surface while simultaneously generating an antithrombogenic releasate under trauma conditions. Traumatization of ECFCs led to significant alterations in their coagulation profile, with distinct effects observed on both their surface properties and the releasates they generated. After 2 hours of traumatization, ECFCs exhibited a pronounced prothrombogenic shift, as evidenced by a reduction in lag time, an increase in endogenous thrombin potential (ETP) and peak thrombin, and a shortened time to peak thrombin (Figure 6Ai-v). This was accompanied by a modest but significant reduction in protein C (PC) activation on the ECFC surface (supplemental Figure 8Ai-ii), suggesting early impairment of the anticoagulant TM-PC pathway. In contrast, the releasates displayed an antithrombogenic profile, prolonging lag time, decreasing ETP and peak thrombin, and extending the time to peak thrombin (Figure 6Bi-v).

Prolonging the traumatization period to 24 hours (Figure 6Ci) further enhanced the procoagulant activity of the ECFC surface, with continued reductions in lag time and time to peak thrombin, as well as increases in ETP and peak thrombin (Figure 6Ci-v). With prolonged traumatization, impairment of the TM-PC axis also became more pronounced, with a substantial decrease in PC activation (supplemental Figure 8Bi-ii). However, the releasates collected at this time point exhibited a different profile than those obtained at 2 hours, now promoting coagulation by shortening both lag time and time to peak thrombin while leaving ETP and peak thrombin unchanged (Figure 6Di-v). These findings highlight a dynamic, time-dependent response to trauma, in which ECFCs develop a prothrombogenic surface while their releasates initially counteract and later promote TG.

### In vitro traumatization of ECFCs induces global hemostatic changes resembling those observed in trauma patients

**Figure 7.**
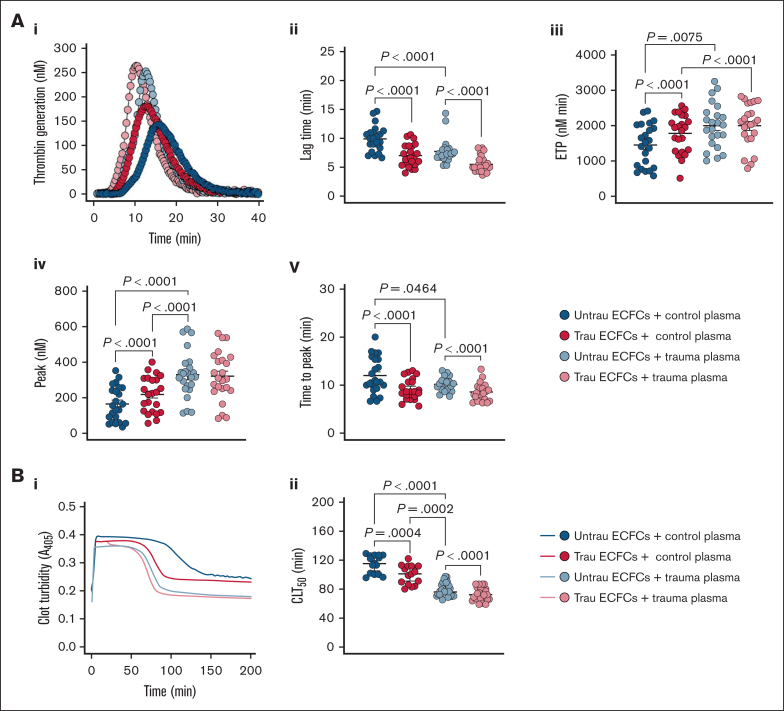
**In vitro traumatization of healthy ECFCs induces global hemostatic changes in healthy plasma resembling those observed in plasma from trauma patients.** (A) TG in control (biological replicate, N = 10; technical replicate, n = 2) or trauma plasma (biological replicate, N = 10; technical replicate, n = 2) over time in the presence of healthy ECFCs cultured under untrau or trau conditions for 2 hours (i), with derived TG parameters: lag times (ii), ETP (iii), peak thrombin (iv), and times to peak thrombin (v). (B) Clot formation and lysis in control (biological replicate, N = 5; technical replicate, n = 3) or trauma plasma (biological replicate, N = 10; technical replicate, n = 3) over time in the presence of healthy ECFCs cultured under untrau or trau conditions for 2 hours (i), with corresponding CLT_50_ (ii). ECFCs were traumatized for 2 hours to model the early phase of endothelial dysfunction, consistent with the clinical context of the trauma plasma samples, which were collected shortly after hospital admission. Data are presented as median with interquartile range. Statistical analysis was performed using the Wilcoxon test for paired comparisons and the Mann-Whitney test for unpaired comparisons in panels Aii-v,Bii; with *P* value <.05 considered statistically significant.

To evaluate whether our in vitro traumatization model induces hemostatic changes in healthy plasma similar to those observed in trauma patients, we assessed TG and CL in the presence of ECFCs under different conditions. In trauma plasma, TG was already enhanced compared to healthy plasma, as indicated by a shorter lag time and time to peak thrombin, as well as increased ETP and peak thrombin (Figure 7Ai-v). Traumatized ECFCs drove a similar procoagulant shift in healthy plasma, reducing lag time and time to peak thrombin and increasing ETP and peak thrombin, although levels remained below those seen in trauma plasma. In trauma plasma, ECFC traumatization further accelerated TG, particularly by shortening lag time and time to peak thrombin. In terms of CL (Figure 7Bi-ii), trauma plasma exhibited a significantly shorter CLT_50_ than healthy plasma, indicating increased fibrinolysis. Exposure to traumatized ECFCs enhanced fibrinolysis in healthy plasma, shortening CLT_50_ while still maintaining a distinction from trauma plasma. Meanwhile, in trauma plasma, traumatized ECFCs further accelerated CL, reinforcing the hyperfibrinolytic state. These findings suggest that ECFC traumatization promotes a procoagulant shift and enhanced fibrinolysis in healthy plasma, mimicking key features of TIC, while further intensifying hypercoagulability and hyperfibrinolysis in trauma plasma.

## Discussion

In this study, we developed a novel in vitro model of EoT by exposing ECFCs to a combination of trauma-relevant conditions that mimic the pathophysiological events occurring during trauma. Our findings demonstrate that this model effectively recapitulates key aspects of EoT, including surface damage, transcriptional reprogramming, hemostatic surface remodeling, and dysregulated coagulation and fibrinolysis. By using these trauma-mimicking factors, we were able to closely simulate the multifaceted hemostatic endothelial responses to trauma, providing critical insights into the endothelial contributions to TIC. Furthermore, this model establishes a robust platform for future mechanistic investigations into the molecular pathways involved in trauma-related endothelial injury and dysfunction.

Many in vitro trauma models focus on isolated stimuli such as hypoxia, shear stress, or single mediators. In contrast, our model integrates multiple trauma-relevant factors, enabling a more physiologically relevant analysis of the endothelial-hemostasis interface in TIC. We used ECFCs over traditional vessel-derived cells, such as HUVECs, due to their unique advantages. We have demonstrated that ECFCs closely mimic HUVECs in hemostatic surface and releasate profiles, rendering them highly suitable surrogates for investigations into endothelial (dys)regulation of hemostasis. Moreover, their derivation from peripheral blood allows for noninvasive sampling and supports inclusion of diverse donors, facilitating investigation of interindividual variability, an important aspect of TIC pathophysiology. Although not tissue specific, ECFCs are broadly applicable and well suited for initial mechanistic studies. Future work should explore endothelial responses in tissue-specific models, such as pulmonary or cerebral endothelium, to capture organ-level nuances in trauma responses.

Our results indicate that EoT after traumatization is a progressive process, beginning with early glycocalyx degradation and culminating in fibrinolytic dysregulation. The significant increase in sTM and syndecan-1 levels after 2 hours suggests rapid endothelial injury, potentially mirroring the early glycocalyx shedding observed in trauma patients.^15^^,^^28^ This degradation compromises vascular integrity and hemostatic balance. Initially, tPA levels increased without a corresponding rise in PAI-1, suggesting an early shift toward fibrinolysis. However, by 24 hours, both tPA and PAI-1 levels were markedly elevated, indicating a dysregulated fibrinolytic response. The persistent elevation of glycocalyx shedding markers further reinforces the notion of sustained endothelial damage, which may contribute to the prothrombotic and hyperfibrinolytic states characteristic of TIC. These findings highlight the time-dependent nature of endothelial injury and suggest that early endothelial damage may predispose to later hemostatic abnormalities.

The transcriptional response to trauma also exhibited distinct early- and late-phase changes. Within 2 hours, ECFCs upregulated inflammatory genes, along with activation of NF-κB signaling and leukocyte adhesion pathways. This suggests that ECs rapidly initiate proinflammatory mechanisms in response to trauma. Simultaneously, the downregulation of genes involved in mitochondrial transport and transcriptional regulation hints at an emerging metabolic shift, likely reflecting early cellular stress. By 24 hours, the transcriptional response became more pronounced, with a substantial increase in differentially expressed genes. The upregulation of stress-related and apoptotic pathways, including endoplasmic reticulum stress and intrinsic apoptotic signaling, indicates a transition from an acute inflammatory response to sustained cellular dysfunction. The emergence of epigenetic regulatory processes, such as histone modification and chromatin remodeling, suggests that prolonged traumatization induces lasting alterations in endothelial function, as has previously been reported in circulating leukocytes.^38^^,^^39^ Meanwhile, the suppression of mitochondrial activity, ribosome biogenesis, and translational processes further supports the notion that sustained trauma disrupts endothelial homeostasis. Collectively, these findings indicate that the endothelial response to trauma evolves over time, progressing from early inflammatory activation to metabolic suppression and transcriptional reprogramming, which may underpin the development of EoT.

The hemostatic profile of traumatized ECFCs also demonstrated a dynamic shift, with an initial procoagulant response followed by fibrinolytic dysregulation. The early downregulation of *THBD* and upregulation of *F3* within 2 hours suggest a rapid endothelial shift favoring TG and clot formation. However, minimal changes in fibrinolysis-associated genes at this stage suggest that early transcriptomic alterations predominantly drive coagulation rather than fibrinolysis. By 24 hours, a more complex pattern emerged, with increased expression of the antifibrinolytic gene *SERPINE1* and downregulation of the profibrinolytic gene *ANXA2*, suggesting a shift toward clot stabilization and impaired fibrinolysis. The sustained suppression of key anticoagulant transcripts, including *THBD* and *SDC1*, reinforces the prolonged prothrombotic state. These findings demonstrate a novel mechanism in which endothelial trauma leads to an evolving hemostatic imbalance through transcriptional dysregulation, potentially contributing to TIC by promoting persistent hypercoagulability and impaired fibrinolysis.

At the protein level, traumatized ECFCs exhibited a progressive prothrombotic shift, characterized by sustained loss of key anticoagulant surface proteins (TM, syndecan-1, and TFPI) and upregulation of the procoagulant TF. The early depletion of these anticoagulant surface proteins, observed within 2 hours, suggests a rapid endothelial response favoring TG and clot formation. These changes became more pronounced over time, with further depletion of anticoagulant surface factors and increased TF expression after 24 hours, reinforcing the hypercoagulable phenotype. The persistent nature of these alterations suggests that endothelial dysfunction in trauma is not merely a transient response but may contribute to sustained coagulopathy. Given the central role of the endothelium in maintaining hemostatic balance,^7^ these findings highlight the potential for targeting endothelial anticoagulant pathways to mitigate TIC and its associated thrombotic complications.

The modulation of fibrinolysis by traumatized ECFCs was also time dependent, with their surface becoming increasingly profibrinolytic while their releasate transitioned toward an antifibrinolytic profile. At 2 hours, ECFCs modestly enhanced CL, primarily through surface-associated mechanisms, whereas their releasate had no measurable effect. However, by 24 hours, the ECFC surface exerted a stronger profibrinolytic effect, whereas the releasate displayed a pronounced antifibrinolytic shift, dramatically prolonging CL time. This delayed accumulation of antifibrinolytic factors, such as PAI-1, suggests a compensatory response aimed at stabilizing clots during prolonged trauma exposure. Notably, the suppression of fibrinolysis observed after 24 hours may contribute to the hypofibrinolysis commonly observed in trauma patients,^40^^,^^41^ further exacerbating the prothrombotic environment. These findings underscore the complex regulation of fibrinolysis by the endothelium and suggest that targeting both surface and secreted factors may be necessary to correct fibrinolytic imbalances in TIC. Moreover, the ECFC releasate exhibits a distinct functional phenotype from the cell surface, shaped by both overlapping and independent regulatory mechanisms. Because its components can disseminate systemically, the releasate may influence fibrinolytic activity at distant sites not directly affected by trauma, potentially contributing to broader dysregulation of coagulation in TIC.

Additionally, ECFC traumatization altered TG in both their surface-associated state and their releasate. At 2 hours, traumatized ECFCs exhibited a procoagulant surface that accelerated and augmented TG, whereas their releasate exerted an opposing anticoagulant effect, suggesting an early compensatory mechanism to counteract excessive coagulation. However, by 24 hours, this balance shifted; the releasate no longer exhibited an anticoagulant effect but instead promoted TG by accelerating its kinetics. This suggests that prolonged trauma exposure overwhelms initial compensatory mechanisms, reinforcing a persistent procoagulant state. The procoagulant shifts in ECFC surface properties were associated with observed reductions in PC activation, suggesting a time-dependent decline in anticoagulant function, likely due to surface TM downregulation and shedding on the ECFC surface. Given the critical role of the PC pathway in regulating coagulation and preventing excessive TG,^42^ this impairment could partly mediate or exacerbate the hypercoagulability observed in trauma. The evolving nature of these changes highlights the dynamic endothelial response to trauma and underscores the complex interplay between surface and secreted factors in TIC.

Finally, our model demonstrated that traumatized ECFCs significantly altered TG and CL in healthy plasma, inducing a hemostatic profile resembling trauma plasma. The acceleration and enhancement of TG suggest that ECFCs induce a hypercoagulable state in healthy plasma, similar in nature but differing in magnitude from that observed in trauma plasma, suggesting that EoT may not be the sole contributor to TIC. Interestingly, CL was also expedited, suggesting a concurrent increase in fibrinolytic activity, which aligns with the hypercoagulable yet hyperfibrinolytic state often observed in severely injured patients.^27^^,^^43^^,^^44^ The exacerbation of both hypercoagulability and hyperfibrinolysis in trauma plasma after ECFC traumatization suggests that EoT may also actively amplify TIC dynamics. Taken together, these findings highlight the model’s clinical relevance and EoT’s pivotal role in trauma-related hemostatic imbalance.

Although our in vitro model successfully captures key aspects of trauma-induced endothelial dysfunction, it has inherent limitations. The simplified culture conditions do not fully replicate the complex in vivo environment, including interactions with immune cells, platelets, and shear stress effects. Additionally, although the concentrations used for each mediator were informed by trauma literature, we recognize that they represent a snapshot and may not reflect the full dynamic or interpatient range. Notably, HMGB1 levels can vary widely across individuals and time points after injury and is among numerous other DAMPs (eg, histone H4^45^^,^^46^ and mitochondrial DNA^47^^,^^48^) whose levels rise after traumatic injury. Future studies should integrate coculture systems, microfluidic approaches, and other trauma-relevant mediators to better mimic pathophysiological conditions. Despite these limitations, our findings provide valuable mechanistic insights and establish a foundation for future translational research. The model can be leveraged to test potential therapeutic interventions targeting endothelial dysfunction in trauma, paving the way for novel strategies to mitigate TIC and improve patient outcomes.

In conclusion, our study demonstrates that ECFCs exposed to trauma-relevant conditions faithfully replicate key features of EoT and TIC observed in patients, capturing the dynamic, time-dependent nature of endothelial injury and its interactions with hemostatic processes. Our model provides a valuable platform for investigating the endothelial mechanisms underlying TIC and offers new opportunities for developing targeted therapeutic strategies aimed at modulating coagulation and fibrinolysis in trauma patients.

Conflict-of-interest disclosure: The authors declare no competing financial interests.

## Supplementary Material

Supplemental Methods, Tables, Figures, and References
