# Supplementary material for: A novel in vitro model of trauma-induced endotheliopathy provides a platform for studying mechanisms of coagulopathy
Source: Blood Vessel Thromb Hemost. 2025 Jul 5;2(4):100087. doi: 10.1016/j.bvth.2025.100087 (PMC12446524; doi:10.1016/j.bvth.2025.100087)

Supplemental material

Supplemental methods

Cell culture. ECFC cultures were established as previously described[1,2]. Citrated peripheral bloods were collected from 6 healthy donors after donor consent and with Wales research ethics committee approval (REC Reference: 20/WA/0313; **Supplemental Table 1**). Bloods were diluted with an equal volume of PBS (Gibco), gently layered on 15 ml of Ficoll-Paque Plus (GE Healthcare) and centrifuged at 1000*g* for 20 min with both the accelerator and decelerator (break) turned off. The buffy coat layer was aspirated, mixed with PBS, and centrifuged at 540*g* for 7 min to pellet the peripheral blood mononuclear cells (PBMCs). PBMCs were then resuspended in endothelial cell growth medium 2 (EGM2; Lonza) supplemented with 20% foetal bovine serum (FBS; Gibco) and centrifuged at 540*g* for 10 min. PBMCs were resuspended at 700,000 cells/ml and seeded in 6-well plates precoated with rat tail collagen type I (Corning) at 5 μg/cm^2 in 0.02 M acetic acid (Merck Millipore) for 1.5 hr at 37°C to allow for collagen crosslinking. First media change was performed after 48 hr and then every 3 days until ECFC colonies appeared between 14 and 28 days. Once the colonies grew to a diameter > 1 mm, they were detached with 0.25% trypsin-EDTA (Gibco), expanded in T25 flasks and cryopreserved in FBS containing 10% (v/v) DMSO. For clot lysis, thrombin generation and protein C activation assays, ECFCs were seeded in 96-well plates at 10,000 cells/well and incubated for 48 hr. Prior to performing the assays, ECFCs were washed twice with PBS. For RNA and protein extraction and culture supernatant collection, ECFCs were seeded in 6-well plates at 100,000 cells/well and grown to confluence.

Pooled human umbilical vein endothelial cells (HUVECs) were purchased from Lonza and cultured in EGM2 (Lonza) according to the supplier's instructions. For RNA and protein extraction, HUVECs were seeded in 6-well plates at 100,000 cells/well and grown to confluence.

***In vitro* traumatisation of ECs.** HUVECs or ECFCs were treated with a cocktail of trauma-related factors at concentrations within ranges measured in trauma patients, including 1 nM epinephrine[3-5] (Sigma-Aldrich), 0.1 ng/ml TNFα[6] (R&D Systems), 0.1 ng/ml IL-6[7,8] (R&D Systems), 500 ng/ml HMGB1[9,10] (Abcam) and 10 μM H_2O_2 (**Supplemental Table 2**), and incubated for 2 and 24 hr in a hypoxia chamber with 1% O_2. These conditions mimic the sympathoadrenal activation, inflammation, tissue damage-induced release of DAMPs and H_2O_2[11], and tissue hypoperfusion/hypoxia that occur in response to traumatic injuries. HUVECs or ECFCs were also treated with buffers used to reconstitute the recombinant proteins (i.e., PBS) and incubated for 2 and 24 hr in a standard, humidified CO_2 incubator with ~20% O_2.

Immunocytochemistry. Cells were seeded at a density of 15,000 cells per chamber in Nunc Lab-Tek 8-chamber slides (ThermoFisher Scientific). After reaching confluence, cells were washed thrice with PBS and fixed in 4% paraformaldehyde in PBS (Santa Cruz Biotechnology) for 15 min. Fixed cells were washed thrice with PBS to remove excess PFA and permeabilised with PBS with 0.1% Triton X-100 for 5 min. Permeabilised cells were washed thrice with PBS and blocked with 10% Normal Goat Serum (derived from the same species as the secondary antibody) in PBS (ThermoFisher Scientific) for 1 hr at room temperature. Cells were incubated with primary antibodies (**Supplemental Table 3**) in Antibody Diluent (Dako) at 4°C overnight. After incubation with primary antibodies, cells were washed thrice with PBS and incubated with fluorescent secondary antibodies (**Supplemental Table 3**) and Alexa Fluor 647-Phalloidin (Invitrogen; 1: 300) in Antibody Diluent in the dark for 1 hr at room temperature. Cells were washed thrice with PBS and mounted with ProLong Gold Antifade Mountant containing the nuclear stain DAPI (Invitrogen). Cells were imaged using a Leica TCS SP8 confocal laser scanning microscope.

Flow cytometry. Cultured HUVECs or ECFCs were detached using 0.25% trypsin-EDTA and resuspended in Cell Staining Buffer (Biolegend) at 5,000,000 cells/ml. 500,000 cells were pre-incubated with Human TruStain FcX (Biolegend) for 10 min at room temperature (RT) to block the Fc receptors. Cells were then incubated with fluorophore-conjugated antibodies or isotype controls (**Supplemental Table 5**) for 20 min on ice in the dark. Cells were washed twice with Cell Staining Buffer and centrifugation at 350*g* for 5 min and stained with live/dead dye 7-AAD (Biolegend) for 5 min on ice in the dark. Flow cytometry was performed on the BD LSRFortessa X20 Cell Analyser (BD Biosciences), capturing 10,000 events, and data were analysed using the FlowJo software (v10.10). Following doublet and dead cell exclusion, cells were assessed for surface expression of CD45, CD34, CD31, CD144 (VE-cadherin), CD309 (VEGFR2) and CD141 (TM).

Protein extraction. Cells were washed thrice with ice-cold PBS and lysed in radioimmunoprecipitation assay (RIPA) buffer (Sigma-Aldrich) supplemented with cOmplete protease inhibitor cocktail (Roche) and phosphatase inhibitor cocktails 2 and 3 (Sigma-Aldrich). Lysates were centrifuged at 600*g* for 10 min and supernatants were collected and stored at -80°C until used. Pierce BCA Assay (ThermoFisher Scientific) together with Pierce BSA standards (0-2000 µg/ml) were used to determine the total protein concentrations of the lysates as recommended by the manufacturer. Optical density was measured at 560 nm using a Multiskan Ascent microplate reader (ThermoScientific).

Immunoblotting. Proteins were boiled in NuPAGE LDS Sample Buffer (Invitrogen) and NuPAGE Sample Reducing Agent (Invitrogen) at 70°C for 10 min. Equal amounts of protein (20 µg) along with SeeBlue Plus 2 Pre-stained Protein Standards (Invitrogen) were loaded into the wells of NuPAGE 4-12% Bis-Tris Protein Gels (Invitrogen) and run in NuPAGE MOPS SDS Running Buffer (Invitrogen) at 160 V for 50 min. Proteins were then transferred to nitrocellulose membranes (Amersham) in NuPAGE Transfer Buffer (Invitrogen) with 20% methanol at 30 V for 2 hr. Successful transfer was confirmed using Ponceau S solution (Sigma-Aldrich), which was removed by washing with PBS with 0.1% Tween 20 (PBST; Sigma-Aldrich) for 5 min with agitation. Membranes were blocked in Tris-buffered saline (TBS) with 0.1% Tween 20 (TBST; Cell Signalling) and 5% skimmed milk (Marvel) for 1 hr. After blocking, membranes were washed thrice with PBST for 5 min each to remove excess blocking buffer and incubated with the primary antibodies (**Supplemental Table 6**) diluted in TBST with 5% BSA (Sigma-Aldrich) at 4°C overnight with rolling. After incubation with primary antibodies, membranes were washed thrice with PBST for 10 min each and incubated with HRP-linked secondary antibodies (**Supplemental Table 6**) diluted in TBST with 5% BSA for 1 hr at room temperature with rolling. Membranes were washed thrice with PBST for 10 min each before being subjected to a mixture of equal volumes of Amersham ECL Western Blotting Detection Reagents 1 and 2 (GE Healthcare). Uncropped blots with protein standards are shown in **Supplemental Figure 3**.

RNA extraction and purification. Cells were washed thrice with ice-cold PBS and lysed in RLT buffer (Qiagen). Total RNA was extracted and purified from cell lysates using the RNeasy Mini Kit (Qiagen) according to the manufacturer's instructions. The concentration and purity of the RNA eluates were assessed using the NanoDrop 2000 Spectrophotometer (ThermoFisher Scientific). RNA samples with A260/A280 ratios of ~2 were deemed acceptable quality for RT-qPCR.

One-step reverse transcription-quantitative polymerase chain reaction (RT-qPCR). The QuantiNova SYBR Green RT-PCR Kit (Qiagen) together with the RotorGene 6000 (Corbett Research) were used to determine the relative mRNA expression of genes of interest according to the manufacturer's instructions. Threshold cycle (Ct) values were determined for genes of interest (**Supplemental Figure 5**) and the housekeeping genes *TBP*, *GAPDH* and *B2M*. The delta Ct ($2^{-\Delta Ct}$) method was used to determine the mRNA expression of genes of interest (**Supplemental Table 7**) relative to the 3 housekeeping genes.

Bulk RNA sequencing and analysis. Bulk RNA sequencing (RNA-seq) was performed by Novogene. Briefly, mRNA was extracted from total RNA using poly-T oligo-attached magnetic beads. cDNA libraries were prepared using directional and non-directional protocols, followed by end repair, A-tailing, adapter ligation, size selection, and PCR amplification. Libraries were quantified using Qubit and real-time PCR and assessed for size distribution using a bioanalyzer before sequencing on an Illumina platform. Raw RNA-seq reads were processed by removing adapters, low-quality reads, and poly-N sequences. Reads were mapped to the reference genome using Hisat2 v2.0.5. Gene expression was quantified using featureCounts v1.5.0-p3 and normalized as Fragments Per Kilobase of transcript per Million mapped reads (FPKM). Differential expression analysis was performed using DESeq2, with genes meeting an adjusted p-value ≤ 0.05 considered differentially expressed. Gene Ontology (GO) enrichment analysis was conducted using the clusterProfiler R package. Volcano and dot plots were generated using the EnhancedVolcano and ggplot2 R packages.

Enzyme-linked immunosorbent assay (ELISA). Cell culture supernatants were collected from ECFCs and centrifuged at 1000*g* for 10 min to remove cell debris. ELISAs for sTM (undiluted; ab214029; Abcam), PAI-1 (dilution: 1 in 200; ab108891; Abcam), syndecan-1 (undiluted; DY2780; R&D Systems) and tPA (dilution: 1 in 10; ab190812; Abcam) were performed following the manufacturer's instructions.

Clot lysis assay. Clot lysis assay was conducted as previously described[12]. Mixtures of 30% (v/v) CRYOcheck pooled normal plasma (PNP; PrecisionBiologic), 16 µM Rox Phospholipid TGT (Rossix) and 50 pM tPA (Sigma-Aldrich) in Tris-buffered saline (TBS; 10 mM Tris, 140 mM NaCl, pH7.4) were first added. In some cases, platelet-poor plasma from individual healthy donors or patients with traumatic injuries (**Supplemental Table 4**) from the Activation of Coagulation and Inflammation in Trauma (ACIT) study[13] were used in place of PNP. To investigate the effects of cell culture supernatants on clot lysis, PNP was supplemented with 20% (v/v) cell culture supernatant. Clotting was initiated with a mixture of 0.1 U/ml thrombin from human plasma (Sigma-Aldrich) and 10.6 mM $CaCl_2$. Clot formation and lysis were monitored by measuring absorbance at 405 nm at 1-min intervals for 4 hr using a Multiskan Ascent microplate reader (ThermoScientific) and the Ascent Software (v2.6). Times to 50% clot lysis (CLT_{50}) in min were determined using the Shiny app for clot lysis[14].

Thrombin generation assay. Thrombin generation was assessed using the Calibrated Automated Thrombography (CAT) method[15]. Thrombin Calibrator or MP-Reagent (Diagnostica Stago), containing 4 µM phospholipids only, were first added followed by PNP. In some cases, plasma from individual healthy donors or patients with traumatic injuries from the ACIT study[13] were used in place of PNP. To investigate the effects of cell culture supernatants on clot lysis, PNP was supplemented with 20% (v/v) cell culture supernatant. Plate was incubated at 37°C for 10 min prior to the addition of FluCa (Diagnostica Stago), containing $CaCl_2$ and the fluorogenic, thrombin-specific substrate Z-Gly-Gly-Arg 7-amido-4-methylcoumarin. Fluorescence was measured every 30 s for 90 min using a Fluoroskan Ascent Fluorometer (ThermoScientific) in conjunction with the Thrombinoscope software (v5.0, Thrombinoscope BV). This yielded thrombin generation parameters, including lag time in min, endogenous thrombin potential (ETP) in nM·min, peak thrombin in nM and time to peak in min.

Protein C activation assay. PC activation was assessed as previously described[16] with some modifications. 70 nM PC (Sigma-Aldrich) in PBS supplemented with 0.6 mM $MgCl_2$ and 1% (w/v) BSA was first added followed by a mixture of 0.1 U/ml thrombin and 3 mM $CaCl_2$ in PBS. The plate was incubated at 37°C for 30 min. 1 U/ml hirudin (Sigma-Aldrich) in PBS was used to stop the reaction. 0.42 mg/ml BIOPHEN CS-21(66) (HYPHEN BioMed), a chromogenic substrate for aPC, was added and absorbance at 405 nm was measured every 30 s for 2 hr using a Multiskan Ascent microplate reader (ThermoScientific) and the Ascent Software (v2.6). PC activation rates were calculated using the Shiny app for zymogen activation[14].

Cell viability assay. Cell viability was assessed using the PrestoBlue Cell Viability Reagent (Thermo Fisher Scientific) according to the manufacturer's instructions. ECFCs were seeded in 96-well plates at 10,000 cells per well, cultured for 48 hr and treated as described. At the endpoint, culture medium was replaced with fresh medium containing 10% PrestoBlue reagent and incubated for 1 hour at 37°C. Absorbance at 570 nm was measured using a Multiskan Ascent microplate reader (ThermoScientific) and the Ascent Software (v2.6). Viability was expressed as a percentage relative to untreated controls.

Supplemental tables

Supplemental Table 1: Characteristics of ECFCs used in this study and the patients from whom they were isolated.

ECFC	Age	Sex	Number of colonies	Time to colony appearance (days)
**Controls**				
1	49	Female	4	16
2	26	Female	3	17
3	34	Male	4	17
4	25	Male	9	9
5	25	Male	3	23
6	30	Male	6	20

Supplemental Table 2: Trauma-relevant factors used for in vitro traumatisation of ECFCs.

Factor	Concentration Used	Reported Clinical Range	Reference(s)	Clinical Relevance
Epinephrine	1 nM	~0.5–2 nM	Ostrowski et al., 2017; Johansson et al., 2012	Sympathoadrenal activation during haemorrhagic shock
TNFα	0.1 ng/mL	~0.01–0.2 ng/mL	Rabinovici et al., 1993	Early pro-inflammatory cytokine released post-injury
IL-6	0.1 ng/mL	~0.01–0.3 ng/mL	Gebhard et al., 2000; Okeny et al., 2015	Key mediator of systemic inflammatory response
HMGB1	500 ng/mL	~5–500 ng/mL	Peltz et al., 2009; Cohen et al., 2009	DAMP released after tissue injury; activates innate immune responses
Hydrogen peroxide (H_2O_2)	10 µM	Not well-defined (localized spikes)	van der Vliet et al., 2014	Mimics oxidative stress and ROS exposure post-trauma
Hypoxia (O_2 level)	1% O_2	N/A (modelled condition)	Standard *in vitro* trauma models	Simulates hypoperfusion/tissue ischemia seen in trauma and shock

Supplemental Table 3: Primary and secondary antibodies used in immunofluorescence.

Antibody	Species	Dilution	Supplier	Catalogue No.
**Primary**				
TM	Mouse	1:500	Abcam	ab6980
CD31	Mouse	1:100	Dako	M0823
vWF	Rabbit	1:200	Dako	A0082
Syndecan-1	Rabbit	1:200	Abcam	ab128936
TF	Rabbit	1:100	Abcam	ab228968

TFPI	Rabbit	1:100	Abcam	ab180619
IgG1 kappa isotype control	Mouse	1:250 - TM<br>1:248 - CD31	Invitrogen	14-4714-82
IgG polyclonal isotype control	Rabbit	1:357 - vWF<br>1:181 - syndecan-1<br>1:192 - TF<br>1:147 - TFPI	Abcam	ab37415
**Secondary**				
Anti-mouse IgG, DyLight 488-linked	Goat	1:250	Invitrogen	35502
Anti-rabbit IgG, Alexa Fluor 488-linked	Goat	1:500	Invitrogen	A-11008

Supplemental Table 4: Demographics and clinical characteristic of trauma patients whose plasma were used in **Figure 7**. Due to the limited availability of plasma, 10 of the 15 patients were used for CL assays and 10 for TG assays.

Clinical characteristic	Trauma patients (*n* = 15)
Age, mean (SD)	40 (20)
ISS, median (IQR)	14 (4-25)
Male, n (%)	12 (80%)
Blunt injury, n (%)	15 (100 %)
GCS, median (IQR)	15 (14-15)
Time from injury to ED (min), mean (SD)	87 (21)
SBP (mmHg), mean (SD)	133 (26)
HR (BPM), mean (SD)	90 (23)
In receipt of TXA pre-admission, n (%)	2 (13%)
Base excess (mEq/mol), median (IQR)	0.2 (-2.1-2.0)
**Pre-hospital therapy**	
Crystalloid (ml), median (IQR)	0 (0-0)
PRBC (units)	0
FFP (units)	0
**Bloods at admission**	
Hb (g/l), mean (SD)	144 (14)
Platelet count ($\times 10^{-9}$/l), mean (SD)	242 (69)
APTT (sec), median (IQR)	28.9 (22.2-31.0)
INR, median (IQR)	1.0 (0.9-1.0)
Clauss Fg (g/ml), median (IQR)	7445 (2199-19400)

Abbreviations: APTT: activated partial thromboplastin time; ED: emergency department; FFP: fresh frozen plasma; Fg: fibrinogen; GCS: Glasgow coma score; Hb: haemoglobin; HR: heart rate; INR: international normalised ratio; ISS: injury severity score; PRBC: packed red blood cells; SBP: systolic blood pressure; TXA: tranexamic acid.

Supplemental Table 5: Antibodies and isotype controls used in flow cytometry. *Per 500,000 cells.

Antibody	Species	Volume (µl)*	Supplier	Catalogue No.
Alexa Fluor 647 anti-human CD144 (VE-cadherin)	Mouse	2.5	Biolegend	348513
Pacific Blue anti-human CD34	Mouse	2.5	Biolegend	343511
Brilliant Violet 605 anti-human CD141 (TM)	Mouse	2.5	Biolegend	344117
APC/Cyanine anti-human CD45	Mouse	2.5	Biolegend	368515
PE anti-human CD309 (VEGFR2)	Mouse	2.5	Biolegend	393003
FITC anti-human CD31 (PECAM1)	Mouse	5	BD Biosciences	555445

Alexa Fluor 647 Mouse IgG2a κ isotype control	Mouse	20	Biolegend	400234
Pacific Blue IgG1 κ isotype control	Mouse	2.5	Biolegend	400131
Brilliant Violet 605 IgG1 κ isotype control	Mouse	3.75	Biolegend	400161
APC/Cyanine 7 IgG1 κ isotype control	Mouse	0.625	Biolegend	400127
PE IgG1 κ isotype control	Mouse	2.5	Biolegend	393003
FITC IgG1 κ isotype control	Mouse	2.5	Biolegend	981802

Supplemental Table 6: Primary and secondary antibodies used in western blotting.

Antibody	Species	Dilution	Supplier	Catalogue No.
**Primary**				
GAPDH (loading control)	Mouse	1:1,000	Cell Signaling	97166
CD31	Mouse	1:1,000	Dako	M0823
vWF	Rabbit	1:2,000	Dako	A0082
VEGFR2	Rabbit	1:1,000	Cell Signaling	2479
VE-cadherin	Rabbit	1:1,000	Abcam	ab33168
**Secondary**				
Anti-rabbit IgG, HRP-linked	Goat	1:1,000	Cell Signaling	7074
Anti-mouse IgG, HRP-linked	Horse	1:1,000	Cell Signaling	7076

Supplemental Table 7: QuantiTect Primer Assays used in RT-qPCR.

Gene	Species	Supplier	GeneGlobe ID
B2M	Human	Qiagen	QT00088935
GAPDH	Human	Qiagen	QT00079247
TBP	Human	Qiagen	QT00000721
VWF	Human	Qiagen	QT00051975
PECAM1	Human	Qiagen	QT00081172
KDR	Human	Qiagen	QT00069818
CD34	Human	Qiagen	QT00056497
CDH5	Human	Qiagen	QT00013244

Supplemental figures

Supplemental Figure 1: Size, doublet and dead cell exclusion and fluorescence minus one controls to guide gating. (**A**) Events were first gated for size to exclude debris followed by gating to exclude doublets and 4-AAD+ dead cells. Unstained (**B**) and fluorescence minus one controls for TM-BV605 (**C**), CD31-FITC (**D**), CD309-PE (**E**), CD144-AF647 (**F**), CD-45-APC/Cy7 (**G**) and CD34-PB (**H**) guided gating.

Supplemental Figure 2: ECFCs exhibit an endothelial phenotype comparable to HUVECs. (**A**) Transcript levels of *CD31*, *VWF*, *CDH5* and *CD34* genes relative to the housekeeping genes *TBP*, *B2M* and *GAPDH* in pooled HUVECs (N = 1, n = 3) and ECFCs (N = 6, n = 3). Flow cytometric analysis of HUVECs and ECFCs for surface expression of CD45, CD34, CD31, CD309 (VEGFR2), CD144 (VE-cadherin) and TM. Histograms are representative of pooled HUVECs (n = 1) and ECFCs (n = 6). (**C**) Immunoblotting of proteins from pooled HUVECs (n = 1) and ECFCs (n = 6) for CD31, VEGFR2, vWF and VE-cadherin. GAPDH was used as a loading control. (**D**) Representative images of pooled HUVECs and ECFCs immunofluorescently stained for CD31, vWF and nuclei. HUVECs and ECFCs were also stained with Phalloidin to visualise the actin cytoskeleton and reveal the cobblestone morphology that is characteristic of ECs. Scale bar: 100 µm. Data are presented as median with interquartile range.

Supplemental Figure 3: Uncropped western blots for VE-cadherin, VEGFR2, vWF and CD31 in HUVECs and ECFCs. Proteins from pooled HUVECs (n = 1) and ECFCs (n = 6) were immunoblotted for (**A**) VE-cadherin, (**B**) VEGFR2, (**C**) vWF and (**D**) CD31. GAPDH served as a loading control.

Supplemental Figure 4: ECFCs present a surface with haemostatic effects similar to HUVECs. (**A**) Clot formation and lysis over time in PNP on the surfaces of either HUVECs (N = 1, n = 9) or ECFCs (N = 5, n = 3; **i**), yielding times to 50% clot lysis (CLT_{50}; **ii**). (**B**) Substrate-cleaving activity of aPC generated from 70 nM PC over time on the surfaces of HUVECs (N = 1, n = 3) or ECFCs (N = 5, n = 3; **i**) with derived aPC activity rates (**ii**). (**C**) Thrombin generation in PNP over time on the surfaces of HUVECs (N = 1, n = 12) or ECFCs (N = 5, n = 6) accompanied by the parameters, lag time (**ii**), ETP (**iii**), peak (**iv**) and time to peak (**v**). Data are presented as median with interquartile range. Statistical analysis was performed using the Mann-Whitney test for unpaired comparisons in **A(ii)**, **B(ii)** and **C(ii-v)**, with $p < 0.05$ considered statistically significant.

Supplemental Figure 5: ECFCs generate a releasate with haemostatic effects similar to HUVECs. (**A**) Clot formation and lysis in PNP supplemented with the cell culture supernatants of HUVECs (N = 1, n = 6) and ECFCs (N = 5, n = 3; **i**) over time with derived times to 50% clot lysis (CLT_{50}; **ii**). (**B**) Thrombin generation in PNP supplemented with the cell culture supernatants of HUVECs (N = 1, n = 6) and ECFCs (N = 5, n = 2) over time with the associated parameters lag time (**ii**), ETP (**iii**), peak (**iv**) and time to peak (**v**). Data are presented as median with interquartile range. Statistical analysis was performed using the Mann-Whitney test for unpaired comparisons in **A(ii)** and **B(ii-v)**, with $p < 0.05$ was considered statistically significant.

Supplemental Figure 6: Effect of increasing concentrations of each trauma-relevant factor on clot lysis on the surface of ECFCs. Times to 50% clot lysis (CLT_{50}) in PNP on the surface of ECFCs (N = 3, n = 3) treated with increasing concentrations of IL-6, TNFα, HMGB1, epinephrine (Epi) and H_2O_2 for 2 (**A**) and 24 (**B**) hr. Low clinically relevant concentrations were sufficient to elicit a profibrinolytic response. Data are presented as median with interquartile range. Statistical analysis was performed using the Wilcoxon test for paired comparisons in **A** and **B**, with $p < 0.05$ considered statistically significant.

Supplemental Figure 7: *In vitro* traumatisation has no effect on ECFC viability. (**A**) Viability of ECFCs (N = 5, n = 3) cultured under traumatising (trau) conditions for 2 hr relative to untraumatised (untrau) ECFCs. (**B**) Viability of ECFCs (N = 5, n =3) cultured under trau conditions for 24 hr relative to untrau ECFCs. Data are presented as median with interquartile range. Statistical analysis was performed using the Wilcoxon test for paired comparisons in **A** and **B**, with $p < 0.05$ considered statistically significant.

Supplemental Figure 8: *In vitro* traumatisation inhibits PC activation on the surface of healthy ECFCs. (**A**) Substrate-cleaving activity of activated protein C (aPC), generated from 70 nM protein C (PC), over time in the presence of healthy ECFCs (N = 5, n = 3), cultured under untraumatising (untrau) or traumatising (trau) conditions for 2 hr (**i**), with derived aPC activity rates (**ii**). (**B**) Substrate-cleaving activity of aPC, generated from 70 nM PC over time, in the presence of healthy ECFCs (N = 5, n = 3), cultured under untrau or trau conditions for 24 hr (i), with derived aPC activity rates. Data are presented as median with interquartile range. Statistical analysis was performed using the Wilcoxon test for paired comparisons in **Aii** and **Bii**, with $p < 0.05$ considered statistically significant.

References

1. Martin-Ramirez J, Hofman M, van den Biggelaar M, Hebbel RP, Voorberg J. Establishment of outgrowth endothelial cells from peripheral blood. *Nat Protoc*. Sep 2012;7(9):1709-15. doi:10.1038/nprot.2012.093

2. Ormiston ML, Toshner MR, Kiskin FN, et al. Generation and Culture of Blood Outgrowth Endothelial Cells from Human Peripheral Blood. *J Vis Exp*. Dec 23 2015;(106):e53384. doi:10.3791/53384
3. Ostrowski SR, Henriksen HH, Stensballe J, et al. Sympathoadrenal activation and endotheliopathy are drivers of hypocoagulability and hyperfibrinolysis in trauma: A prospective observational study of 404 severely injured patients. *J Trauma Acute Care Surg*. Feb 2017;82(2):293-301. doi:10.1097/TA.0000000000001304
4. Ostrowski SR, Johansson PI. Endothelial glycocalyx degradation induces endogenous heparinization in patients with severe injury and early traumatic coagulopathy. *J Trauma Acute Care Surg*. Jul 2012;73(1):60-6. doi:10.1097/TA.0b013e31825b5c10
5. Johansson PI, Stensballe J, Rasmussen LS, Ostrowski SR. High circulating adrenaline levels at admission predict increased mortality after trauma. *J Trauma Acute Care Surg*. Feb 2012;72(2):428-36. doi:10.1097/ta.0b013e31821e0f93
6. Rabinovici R, John R, Esser KM, Vernick J, Feuerstein G. Serum tumor necrosis factor-alpha profile in trauma patients. *J Trauma*. Nov 1993;35(5):698-702. doi:10.1097/00005373-199311000-00008
7. Gebhard F, Pfetsch H, Steinbach G, Strecker W, Kinzl L, Brückner UB. Is interleukin 6 an early marker of injury severity following major trauma in humans? *Arch Surg*. Mar 2000;135(3):291-5. doi:10.1001/archsurg.135.3.291
8. Okeny PK, Ongom P, Kituuka O. Serum interleukin-6 level as an early marker of injury severity in trauma patients in an urban low-income setting: a cross-sectional study. *BMC Emerg Med*. Sep 16 2015;15:22. doi:10.1186/s12873-015-0048-z
9. Peltz ED, Moore EE, Eckels PC, et al. HMGB1 is markedly elevated within 6 hours of mechanical trauma in humans. *Shock*. Jul 2009;32(1):17-22. doi:10.1097/shk.0b013e3181997173
10. Cohen MJ, Brohi K, Calfee CS, et al. Early release of high mobility group box nuclear protein 1 after severe trauma in humans: role of injury severity and tissue hypoperfusion. *Crit Care*. 2009;13(6):R174. doi:10.1186/cc8152
11. van der Vliet A, Janssen-Heininger YM. Hydrogen peroxide as a damage signal in tissue injury and inflammation: murderer, mediator, or messenger? *J Cell Biochem*. Mar 2014;115(3):427-35. doi:10.1002/jcb.24683
12. Morrow GB, Beavis J, Harper S, Bignell P, Laffan MA, Curry N. Characterisation of a novel thrombomodulin c.1487delC,p.(Pro496Argfs*10) variant and evaluation of therapeutic strategies to manage the rare bleeding phenotype. *Thromb Res*. Jan 2021;197:100-108. doi:10.1016/j.thromres.2020.11.002
13. Müller MC, Balvers K, Binnekade JM, et al. Thromboelastometry and organ failure in trauma patients: a prospective cohort study. *Crit Care*. Dec 25 2014;18(6):687. doi:10.1186/s13054-014-0687-6
14. Longstaff C, fibrinolysis so. Development of Shiny app tools to simplify and standardize the analysis of hemostasis assay data: communication from the SSC of the ISTH. *J Thromb Haemost*. May 2017;15(5):1044-1046. doi:10.1111/jth.13656
15. Hemker HC, Giesen P, Al Dieri R, et al. Calibrated automated thrombin generation measurement in clotting plasma. *Pathophysiol Haemost Thromb*. 2003;33(1):4-15. doi:10.1159/000071636
16. Rehill AM, Leon G, McCluskey S, et al. Glycolytic reprogramming fuels myeloid cell-driven hypercoagulability. *J Thromb Haemost*. Feb 2024;22(2):394-409. doi:10.1016/j.jtha.2023.10.006

Supplemental figures

Supplemental Figure 1

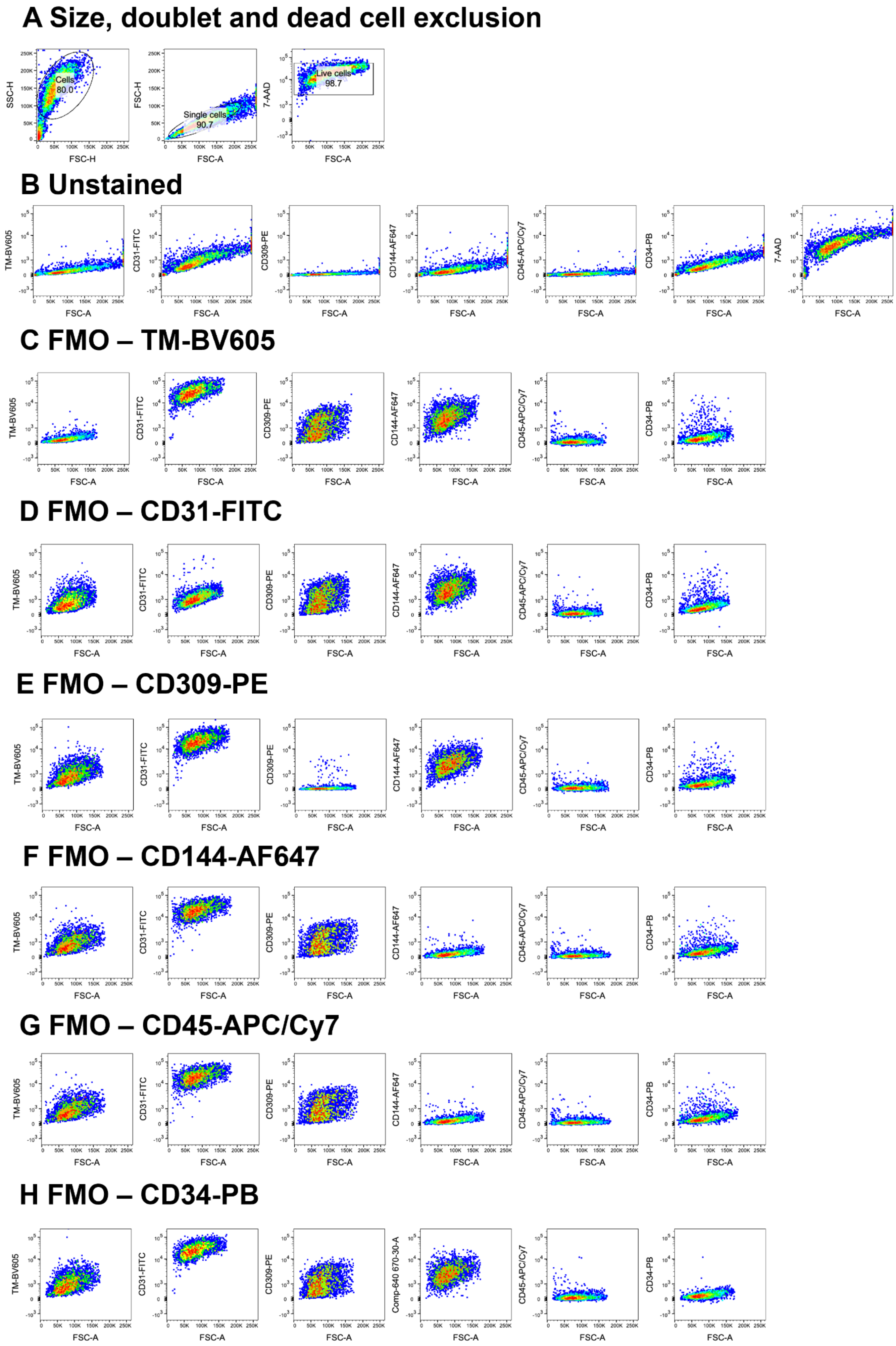

Supplemental Figure 2

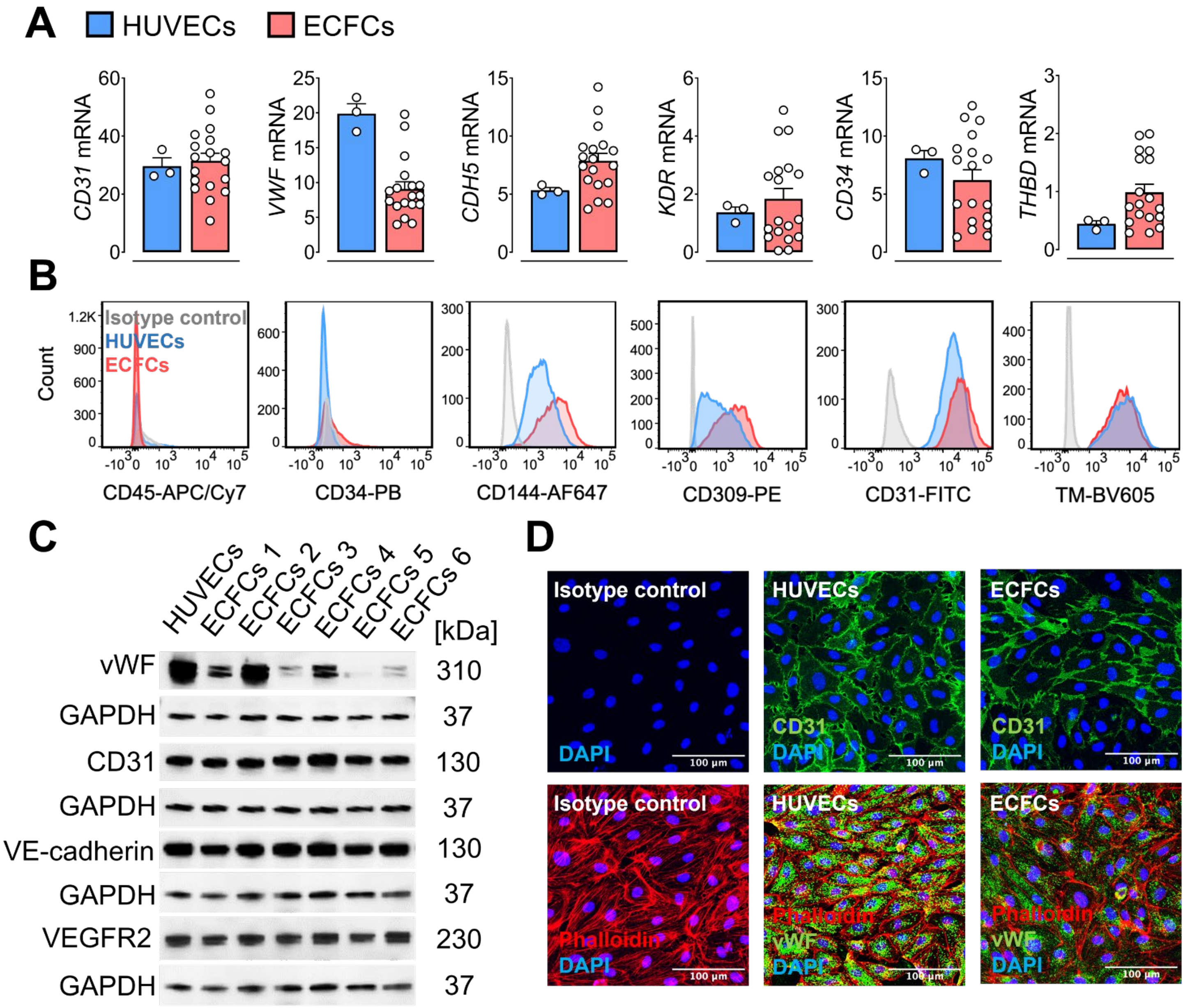

Supplemental Figure 3

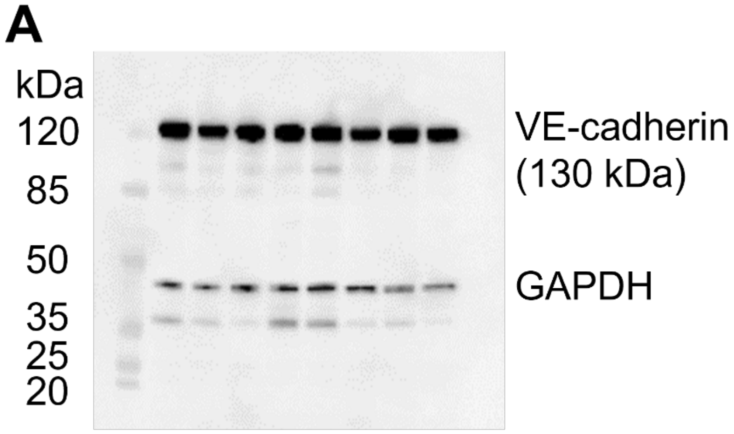

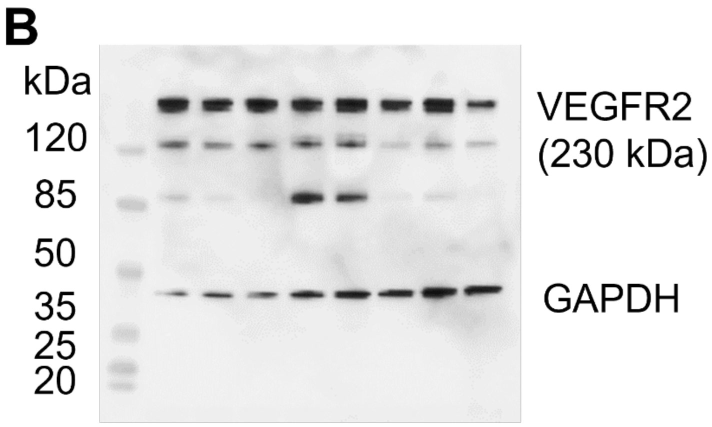

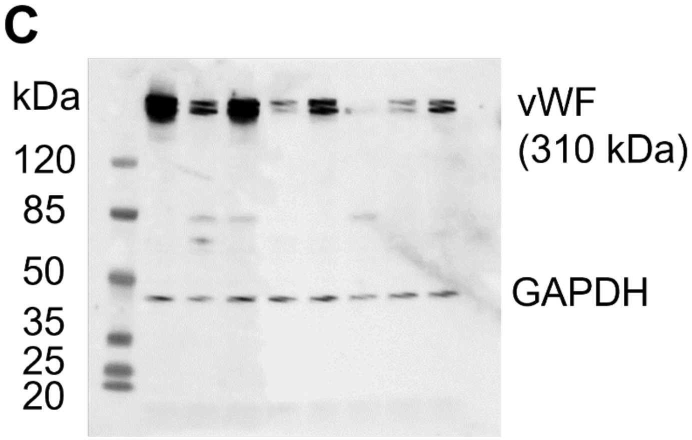

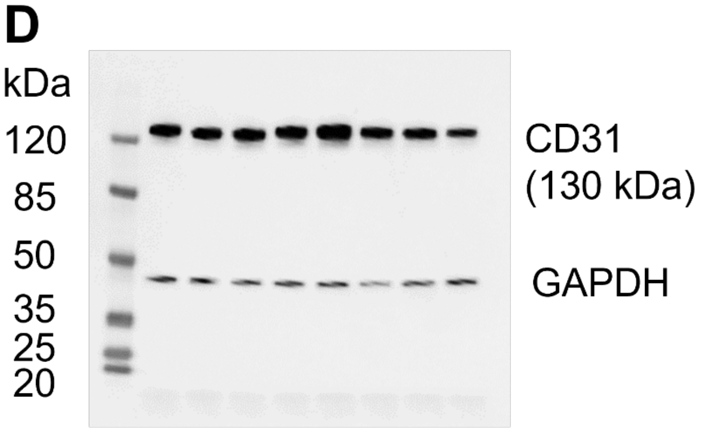

Supplemental Figure 4

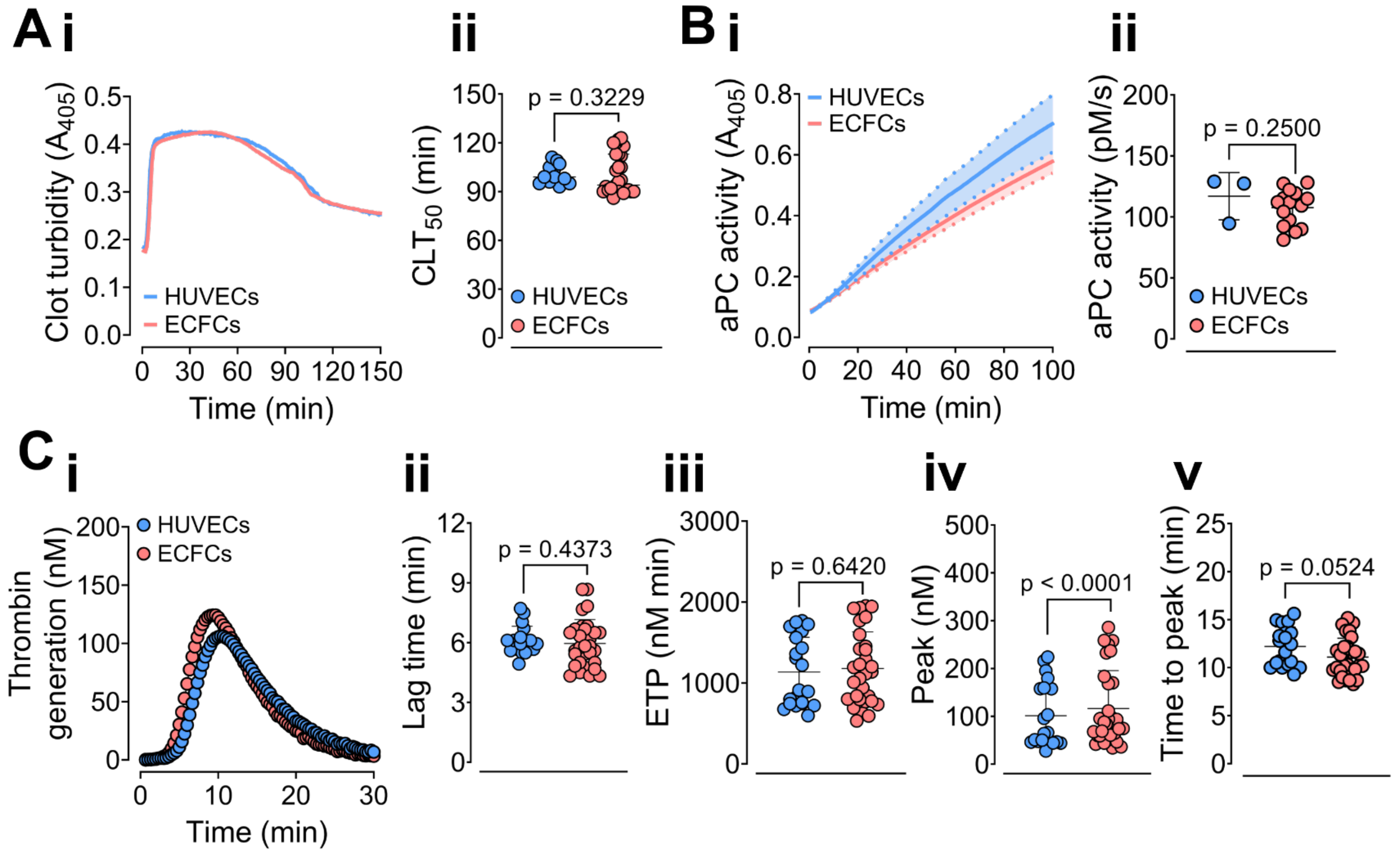

[

Supplemental Figure 5

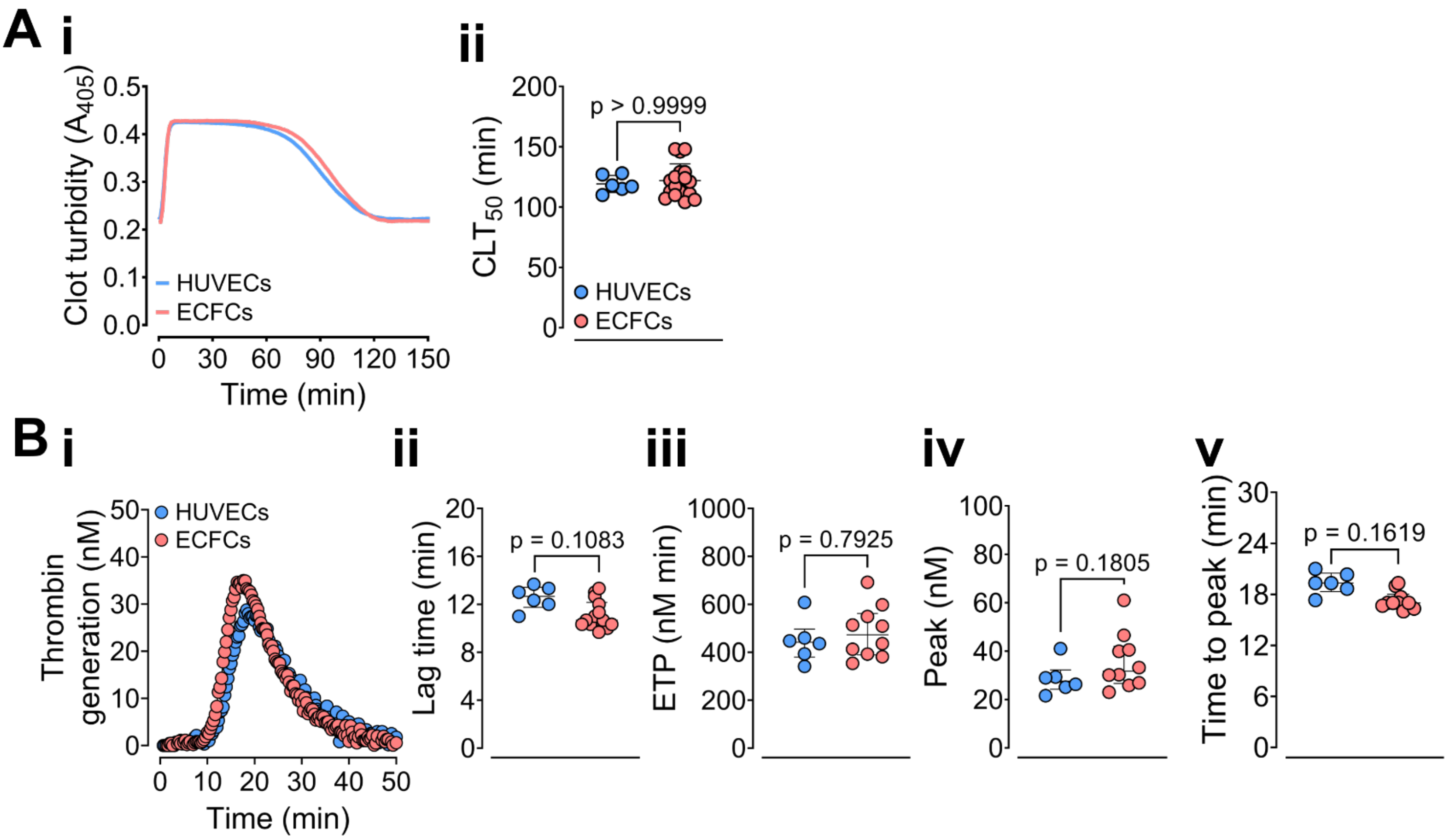

Supplemental Figure 6

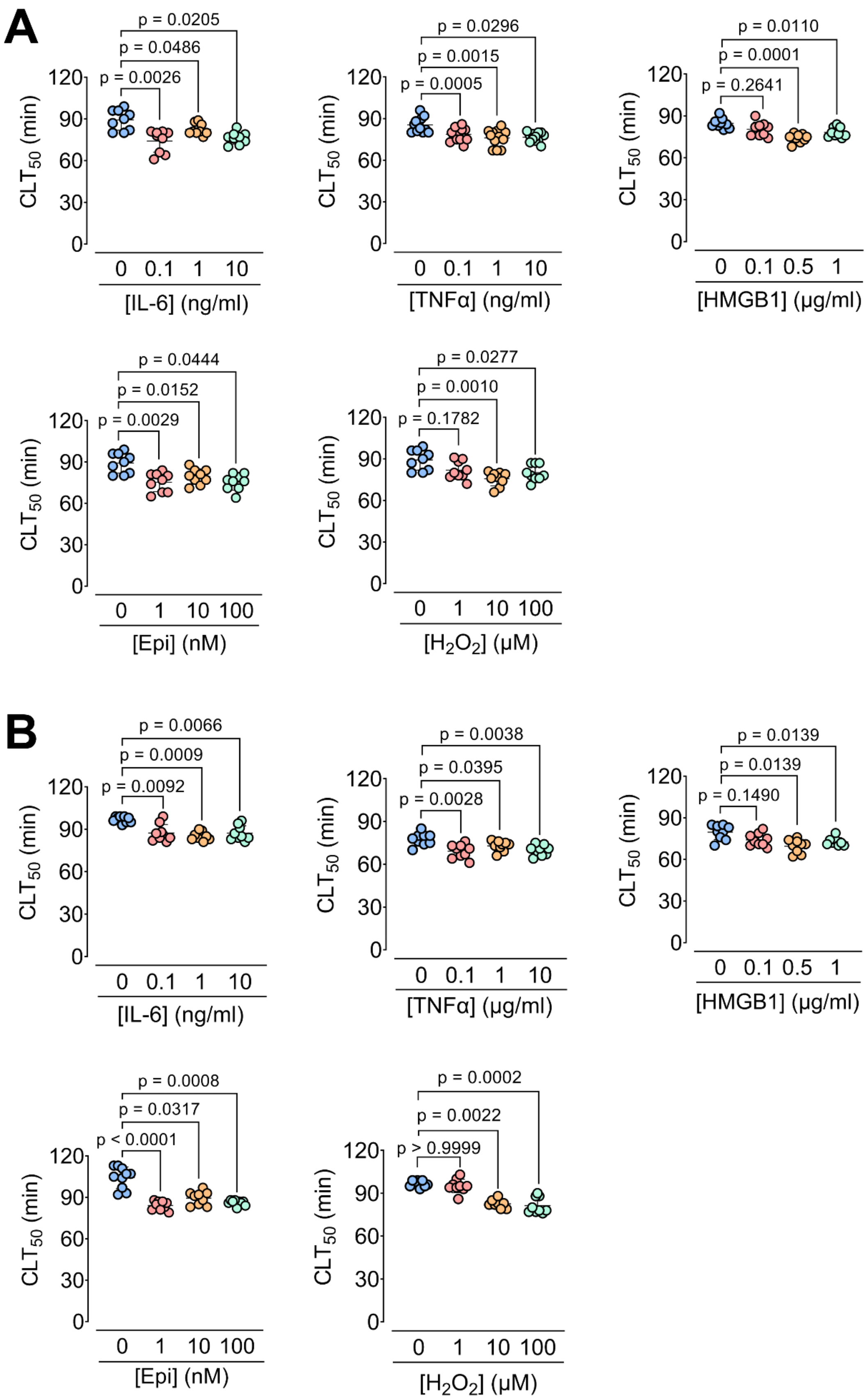

Supplemental Figure 7

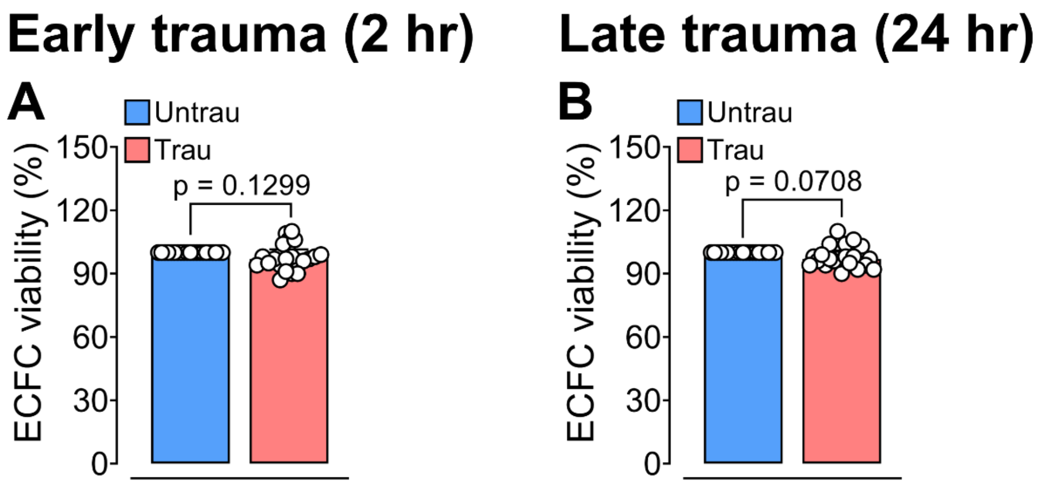

Supplemental Figure 8

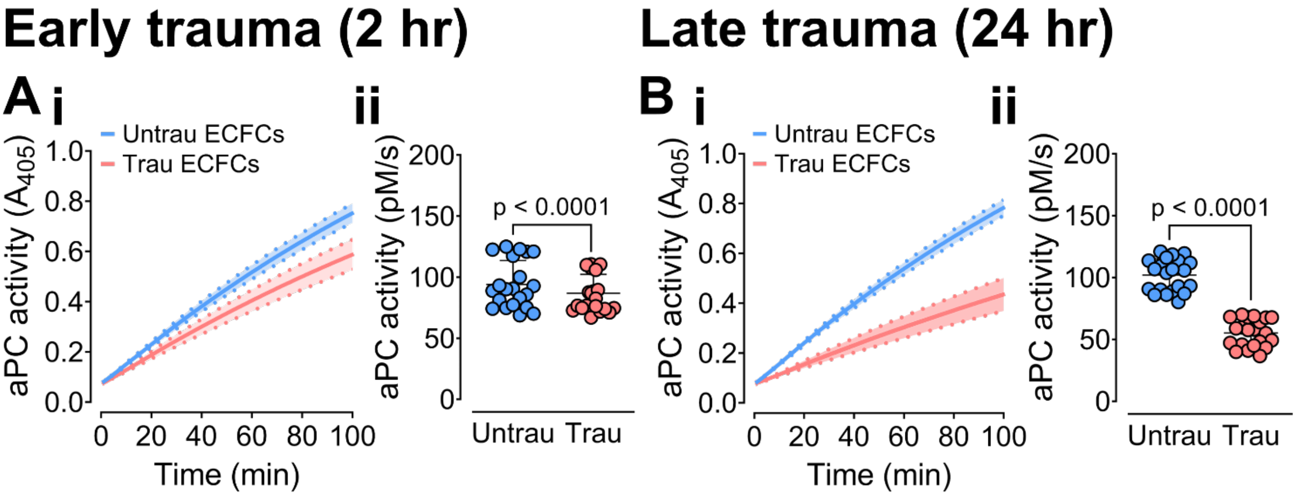

Supplement: Supplemental Methods, Tables, Figures, and References [file BVTH_VTH-2025-000366-mmc1.pdf]
